# ClinCirc identifies alterations of the circadian peripheral oscillator in critical care patients

**DOI:** 10.1172/JCI162775

**Published:** 2023-02-15

**Authors:** Peter S. Cunningham, Gareth B. Kitchen, Callum Jackson, Stavros Papachristos, Thomas Springthorpe, David van Dellen, Julie Gibbs, Timothy W. Felton, Anthony J. Wilson, Jonathan Bannard-Smith, Martin K. Rutter, Thomas House, Paul Dark, Titus Augustine, Ozgur E. Akman, Andrew L. Hazel, John F. Blaikley

**Affiliations:** 1 Faculty of Biology, Medicine and Health, University of Manchester, Manchester, United Kingdom.; 2 Manchester Royal Infirmary, Manchester University NHS Foundation Trust (MFT), Manchester, United Kingdom.; 3 Department of Mathematics, University of Manchester, Manchester, United Kingdom.; 4 Wythenshawe Hospital, MFT, Manchester, United Kingdom.; 5 Northern Care Alliance NHS Foundation Trust (Salford Care Organisation), Salford, United Kingdom.; 6 School of Mathematics, University of Exeter, Exeter, United Kingdom.

**Keywords:** Inflammation, Transplantation, Anesthesiology, Organ transplantation, Translation

## Abstract

**Background:**

Assessing circadian rhythmicity from infrequently sampled data is challenging; however, these types of data are often encountered when measuring circadian transcripts in hospitalized patients.

**Methods:**

We present ClinCirc. This method combines 2 existing mathematical methods (Lomb-Scargle periodogram and cosinor) sequentially and is designed to measure circadian oscillations from infrequently sampled clinical data. The accuracy of this method was compared against 9 other methods using simulated and frequently sampled biological data. ClinCirc was then evaluated in 13 intensive care unit (ICU) patients as well as in a separate cohort of 29 kidney-transplant recipients. Finally, the consequences of circadian alterations were investigated in a retrospective cohort of 726 kidney-transplant recipients.

**Results:**

ClinCirc had comparable performance to existing methods for analyzing simulated data or clock transcript expression of healthy volunteers. It had improved accuracy compared with the cosinor method in evaluating circadian parameters in PER2:luc cell lines. In ICU patients, it was the only method investigated to suggest that loss of circadian oscillations in the peripheral oscillator was associated with inflammation, a feature widely reported in animal models. Additionally, ClinCirc was able to detect other circadian alterations, including a phase shift following kidney transplantation that was associated with the administration of glucocorticoids. This phase shift could explain why a significant complication of kidney transplantation (delayed graft dysfunction) oscillates according to the time of day kidney transplantation is performed.

**Conclusion:**

ClinCirc analysis of the peripheral oscillator reveals important clinical associations in hospitalized patients.

**Funding:**

UK Research and Innovation (UKRI), National Institute of Health Research (NIHR), Engineering and Physical Sciences Research Council (EPSRC), National Institute on Academic Anaesthesia (NIAA), Asthma+Lung UK, Kidneys for Life.

## Graphical abstract

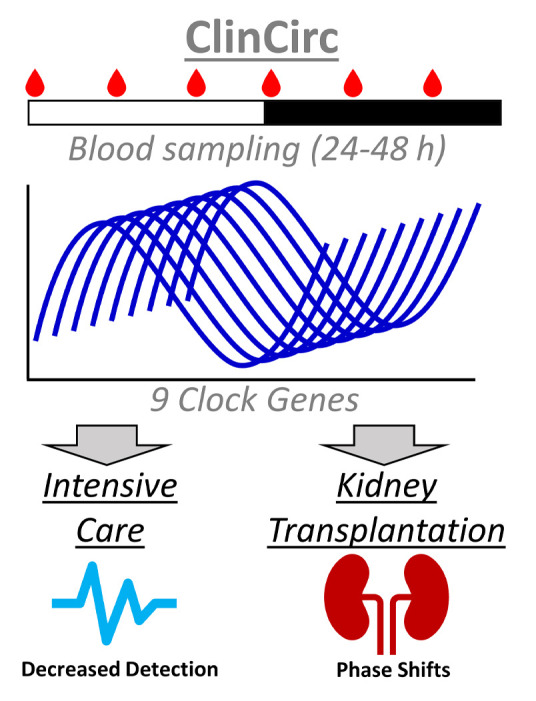

## Introduction

Circadian biology has profound effects on both outcome and pathophysiology (1) in many disease models (2–5). The clinical relevance of these effects, however, remains opaque, since associations between clinical outcomes and the molecular oscillator (6), a key driver of circadian biology, remain scarce. One potential reason for this is the difficulty of determining whether an oscillation is circadian or noncircadian. Therefore, the development of new techniques, or using workflows that combine existing techniques, to identify circadian oscillations with increased accuracy could reveal new insights into the translational importance of circadian biology. In this paper, we adopt the latter approach to create a technique combining the best features from two widely used methods of periodic signal analysis, a technique that we term ClinCirc.

It is possible to measure circadian outputs in order to assess circadian oscillations. However, these outputs may be influenced by noncircadian mechanisms (7, 8), causing them to be subject to confounding. An alternative approach is to measure the molecular oscillator, a core mechanism in circadian biology (9). This oscillator consists of 9 clock proteins regulated by a transcription-translation feedback loop (TTFL). It has 3 defined characteristics: (a) a self-sustained oscillatory period of 24 hours; (b) the period remains constant across a range of temperatures; and (c) its phase can be shifted or “entrained” by exogenous stimuli. The characteristics of this oscillator have been extensively characterized in both animal and human in vitro models (9). In these models, inflammation causes a reduction in amplitude and a shortened period of oscillation (10, 11) potentially mediated via the transcription factor RELA (12). Other stimuli, e.g., corticosteroids, shift the phase of the molecular oscillator through regulation of clock protein (PER and REVERBα) expression (13). In spite of these studies showing that inflammation is associated with alterations in circadian oscillations, this association has not been widely reported in the clinic (14).

A possible reason for the lack of replication in clinical cohorts is a lack of robust techniques for detection given the constraints of clinical sampling. Specifically, samples must be taken relatively infrequently over a short recording window. This is necessary because rapid changes in the clinical status of patients could obscure important clinical associations and adverse effects may arise from an increase in sampling frequency. Therefore, analysis methods must utilize a relatively small number of data points over a short time. In the case of single-step methods, this can be challenging because they have to fit all 4 parameters used to characterize a sinusoidal oscillation (mesor, period, amplitude, phase) at once or make untested assumptions about the parameters, such as assuming that the period is approximately 24 hours. Therefore, existing single-step circadian methods (e.g., χ^2^ periodogram, mFourFit, maximum entropy spectral analysis, and spectrum resampling) are potentially unreliable, as they rely on collection periods lasting several days (15) or high-frequency (minutes to hourly) sampling to make accurate predictions. This caveat also applies to the most widely used method, cosinor analysis, where having sparse data is likely to result in overfitting (16). An alternative is to use machine-learning approaches (e.g., Gaussian process regression [GPR]) (17); however, these are computationally intensive, resulting in long run times. The Lomb-Scargle periodogram (L-SP) is a method for defining the dominant harmonic for an oscillatory signal in both biology (18) and astronomy (19) and has previously been hypothesized to be the most accurate method in this situation. Unfortunately, it does not provide information regarding the 3 remaining characteristics (mesor, amplitude, and phase), making its use in isolation limited in the analysis of circadian rhythms.

A potential solution is to use multiple analysis methods simultaneously to assess circadian rhythms (20, 21). The different methods are often run concurrently rather than sequentially, e.g., CircaN (21) and MetaCycle (22). The final result is determined a posteriori, either by choosing one (best) method or by combining results from multiple methods. We pursued an alternative approach using a sequential workflow in which assumptions about the parameters are tested before proceeding to parametric fitting. Such preprocessing or data-cleaning stages are implicit in many statistical workflows, but have not always been used consistently or been widely applied to measurement of the molecular oscillator in the clinical setting.

The adopted approach, termed ClinCirc, combines L-SP with cosinor analysis in a 2-step sequential method. The L-SP is used as a preprocessing filter to remove noncircadian signals before cosinor analysis is then used to determine the characteristics of detected circadian oscillations. The performance of ClinCirc was then compared against existing methods using simulated data and data from human blood samples (healthy volunteers, critical care patients). On simulated data and healthy volunteers, ClinCirc had comparable sensitivity and specificity when compared with existing methods. In the critical care setting, ClinCirc was the only method tested that revealed that loss of circadian oscillations was associated with inflammation, a feature widely reported in animal models (10–12). Furthermore, after kidney transplantation, in which inflammation is suppressed because of immunosuppression, circadian oscillations were maintained but shifted according to the time of surgery. This shift in circadian phase was associated with adverse outcomes. Therefore, we propose that ClinCirc is a valuable method for identifying the loss of circadian oscillations in clinical cohorts, revealing insights for the translation of circadian biology.

## Results

### The effectiveness of circadian mathematical techniques on simulated data sampled over short sampling periods.

**Figure 1:**
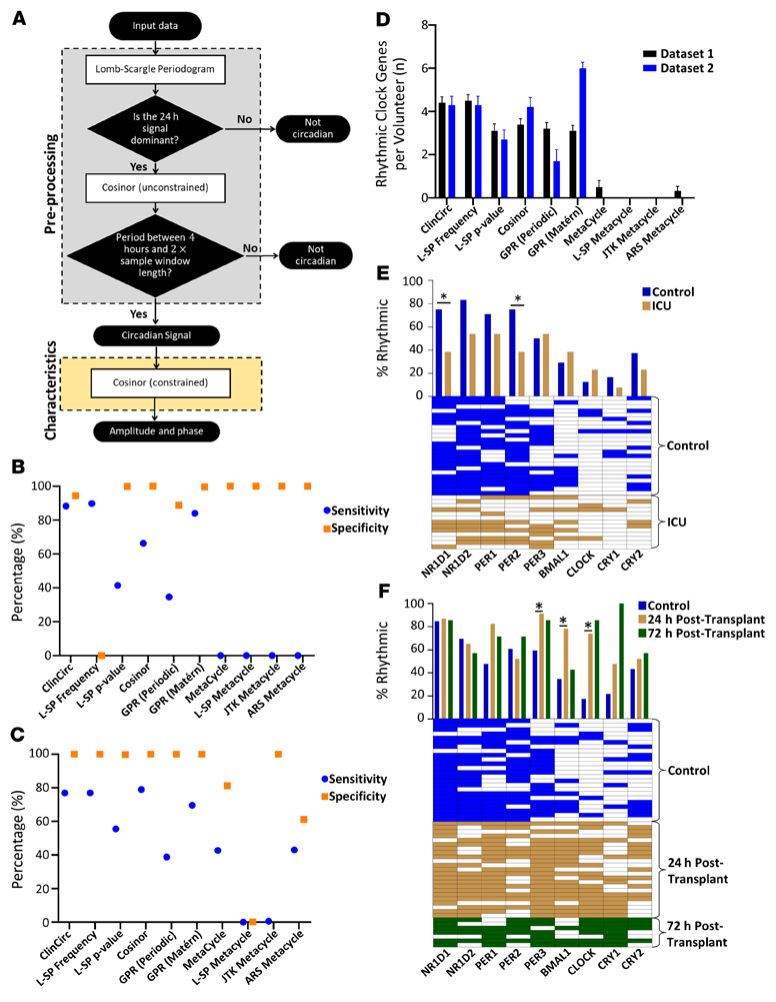
Performance of ClinCirc in detecting circadian rhythmicity. (**A**) Flow diagram depicting how cosinor analysis was combined with L-SP to create ClinCirc. (**B**) Sampling periods of 24 hours and (**C**) 48 hours were used to characterize the sensitivity and specificity of 10 mathematical methods on infrequently (every 4 hours) sampled data. Sensitivity was calculated using a waveform created following the addition of 40% noise (Supplemental Methods) to a sinusoidal wave. Specificity was calculated from a straight line (5,000 simulations). Data are represented as mean ± SD. (**D**) Two data sets measuring clock-gene expression in healthy human volunteers were reanalyzed using the same 10 mathematical methods. The average number of clock genes per volunteer that each method detected as having a circadian rhythm is shown. Data are represented as mean ± SEM. (**E**) ClinCirc was used to evaluate circadian rhythmicity of clock genes in ICU patients (*n* = 13) as well as in healthy volunteers (*n* = 23). Bar chart shows the proportion of subjects in which ClinCirc detected a circadian oscillation per clock gene. Heatmap shows which subjects had a detected circadian oscillation. Each subject is represented by a row, and a filled square represents a detected circadian oscillation. (**F**) ClinCirc was used to evaluate circadian rhythmicity of clock genes in kidney-transplant recipients at 0 to 24 hours after transplantation (*n* = 22; 24 hours after transplant) or 48 to 72 hours after transplantation (*n* = 7; 72 hours after transplant) as well as in healthy volunteers (*n* = 23). Bar chart shows the proportion of subjects that had a circadian oscillation for the clock gene. Heatmap shows which subjects had the circadian oscillation. Each subject is represented by a row in which the filled squares represent a detected circadian oscillation. *P < 0.05, χ^2^ test.

To evaluate the optimal mathematical method for circadian analysis in patients, simulated data were analyzed with 9 existing mathematical techniques. All data had a sampling frequency of 4 hours and were sampled over a period of 24 to 48 hours, replicating the constraints of clinical circadian analysis. In addition to the 9 existing mathematical techniques, we evaluated ClinCirc, a method that combined L-SP with cosinor analysis. In the preprocessing step, L-SP was used to identify the presence of a circadian oscillation by defining the dominant harmonic. An unconstrained cosinor analysis was then used as further preprocessing to exclude cases with excessively high or low periods based on the fit to a single sinusoid. To determine the amplitude and phase of any detected circadian waveform, a cosinor analysis was then used where the period was constrained from 18 to 30 hours (Figure 1A and Supplemental Methods).

To define the sensitivity of the methods, a cosine wave was sampled in the presence and absence of additive noise in all parameters. If the sampling period was 24 hours or 48 hours, then most mathematical methods tested had a sensitivity of 100% on a cosine wave without noise (Supplemental Table 1; supplemental material available online with this article; https://doi.org/10.1172/JCI162775DS1). The exceptions were GPR (Matérn), which had sensitivity of 99.8% over 48 hours, GPR (periodic), which had a sensitivity of 0% over 24 hours or 48 hours, and some of the constituent parts of MetaCycle. The addition of random noise (Supplemental Figure 1) onto the perfect cosine wave caused the methods to have different performances. Over a 24-hour sampling period, ClinCirc, L-SP (frequency), and GPR (Matérn) had the highest sensitivity, greater than 84%, followed by cosinor analysis, with a sensitivity of 66%, and L-SP (*P* value), which had a sensitivity of 41%. GPR (periodic) had a sensitivity of 34.6%, with the remaining methods all having a sensitivity below this (Supplemental Table 1 and Figure 1B). Over a 48-hour sampling period, ClinCirc, L-SP frequency, and cosinor methods all had comparable sensitivities of 76.92%, 76.92%, and 78.94%, respectively (Supplemental Table 1 and Figure 1C). The remaining methods had lower sensitivities, as described in Supplemental Table 1. The specificity of the methods was then evaluated. Over a 24-hour sampling period, this was greater than 88% (Figure 1B) for all methods except for L-SP frequency, which had a specificity of 0%. Over a 48-hour sampling period, specificity was also over 98% for all methods, except for MetaCycle (81%) and two of its constituent parts (L-SP, ARS) (Figure 1C). ClinCirc thus had comparable sensitivity/specificity over both 24 hour and 48-hour sampling periods on simulated data compared with existing methods.

### Accuracy of ClinCirc at estimating amplitude, phase, and period compared with cosinor analysis using in vitro biological data.

In ClinCirc, cosinor analysis was added to the L-SP method to define the characteristics of detected circadian oscillation. To confirm the effectiveness of this approach, bioluminescence traces representative of PER2 expression from PER2:luc lung slices were analyzed at a 4-hour sampling frequency.

The period estimates of ClinCirc were within 2 hours, acrophase estimates were within 1.1 hours, and relative amplitude was within 0.23-fold (Supplemental Figure 2, A–C) of the original data (1 minute sampling frequency). Following the addition of artificial noise (40%), the accuracy of ClinCirc was slightly reduced (Supplemental Figure 2D); however, period estimation was still within 4 hours, acrophase estimation was within 2 hours, and relative amplitude was within 0.5-fold (Supplemental Figure 2, A–C) of the original data. In all cases, ClinCirc analysis was more accurate compared with cosinor analysis of the same data (Supplemental Figure 2D).

### ClinCirc identifies a comparable number of circadian oscillations for clock transcripts in healthy human volunteer data sets compared with cosinor analysis.

The performance of the mathematical methods was then evaluated on two previously published data sets (23, 24) reporting the expression of the molecular oscillator in human peripheral blood. Out of the methods tested, ClinCirc, cosinor, GPR (periodic), and GPR (Matérn) detected similar numbers of circadian oscillations per individual (3–4 oscillations). ClinCirc detected the greatest number of circadian oscillations in data set 1, while GPR (Matérn) detected the most in data set 2 (Figure 1D). The remaining mathematical techniques detected fewer than 2 oscillatory clock transcripts per patient (Figure 1D).

### Study population demographics.

The clinical applicability of ClinCirc was then investigated by analyzing the expression of clock transcripts making up the molecular oscillator in peripheral blood from 2 different patient cohorts. The first cohort of patients were critically ill patients admitted to the intensive care unit (ICU) (Supplemental Table 2). These individuals had varying levels of inflammation and did not receive glucocorticoids, an antiinflammatory medication. This contrasted with our second cohort of patients, kidney-transplant recipients who all received glucocorticoids. This cohort was sampled during their recovery following transplantation (Supplemental Table 3). They were primarily sampled over the first 24 hours following transplantation (24 hours, *n* = 22) with a further 7 being sampled 48 to 72 hours after their surgery.

### Rhythmic oscillator transcripts are reduced in ICU patients, but increased after kidney transplantation.

Initially, the presence or absence of detected circadian oscillations of individual peripheral oscillator transcripts was quantified in patient cohorts. The proportion of ICU patients in whom ClinCirc detected a circadian oscillation was reduced for NR1D1 and PER2 compared with that in healthy volunteers (*P* < 0.05, χ^2^ test; Figure 1E). No differences in the prevalence of detectable circadian oscillations for the remaining 7 clock genes were observed.

Immediately after kidney transplantation, the opposite phenomenon was observed. Patients who had received their kidney transplants within 24 hours had a higher prevalence of detectable circadian oscillations for PER3, BMAL1, and CLOCK compared with healthy volunteers (*P* < 0.05, χ^2^ test; Figure 1F), with no differences being observed for the remaining clock genes. There were no differences in the prevalence of circadian oscillations for the clock genes when kidney recipients at 72 hours were compared with healthy volunteers or kidney recipients at 24 hours.

### Reduction in detectable circadian oscillations is associated with a reduction in inflammatory circadian oscillations.

Patients were split into 2 groups according to whether the detection of circadian oscillations in the molecular oscillator was reduced. A threshold of 3 circadian oscillations for clock genes was chosen since 95% of healthy volunteers fulfilled this criterion (Figure 1D). Seven ICU patients also fulfilled this criterion and were defined as “standard,” while fewer than 3 circadian oscillations were detected in the remaining 6 ICU patients, who were labeled as “reduced”; their clinical characteristics are shown in Supplemental Table 4. For all 29 kidney-transplant recipients, at least 3 circadian oscillations were detected in the molecular oscillator per patient.

**Figure 2:**
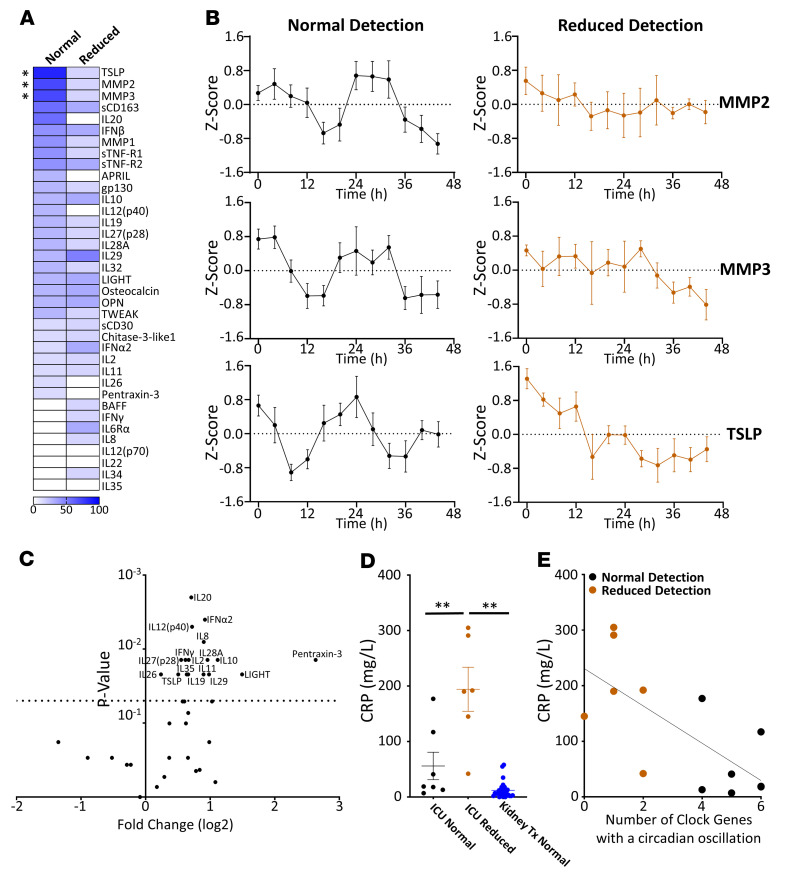
Inflammation is associated with reduced detection of circadian oscillations in the ICU. ICU patients were split into 2 groups based on whether ClinCirc detected unchanged or a reduced number of circadian oscillations in the peripheral blood molecular oscillator. (**A**) Heatmap displaying the proportion of patients in each group in whom ClinCirc detected a circadian oscillation in the measured inflammatory mediator. *P < 0.05, χ^2^ test. (**B**) Forty-eight–hour expression profiles for the 3 inflammatory mediators (MMP2, MMP3, and TSLP) that showed differential circadian oscillations between ICU patients who had standard or reduced detection of circadian oscillations. Data are represented as mean ± SEM. Traces were acrophase aligned. (**C**) Volcano plot showing differences in mean expression of 37 inflammatory mediators between ICU patients with standard or reduced detection of circadian oscillations. Positive fold change reveals that the mediator was elevated in patients in whom detection of circadian oscillations was reduced. Dotted line shows *P* = 0.05. Significant cytokines are labeled. Inflammatory mediators that were differentially regulated are IFN-α2, IFN-γ, IL-2, IL-8, IL-10, IL-11, IL-12 (p40), IL-19, IL-20, IL-26, IL-27 (p28), IL-28A, IL-29, IL-35, LIGHT, Pentraxin-3, and TSLP. (**D**) Difference in CRP expression between ICU patients grouped according to the detection of circadian oscillations and those who underwent kidney transplantation. **P < 0.01, ANOVA post hoc Tukey’s. (**E**) CRP expression in ICU patients was also plotted against the number of clock genes for that participant in which ClinCirc detected the presence of a circadian oscillation. *r^2^* = 0.49 linear correlation. *n* = 13. For circadian rhythm analysis, data were only plotted if a circadian rhythm was detected.

Circadian biology has been widely reported as regulating inflammatory pathways. Therefore, the protein profiles of 37 inflammatory mediators in the plasma were examined to show whether reduced rhythmicity was associated with altered effects. The expression levels of 5 mediators (CD163, IL20, MMP2, MMP3, and TSLP) oscillated in a circadian manner in over 50% of ICU patients with standard detection (Figure 2A). For 3 of these mediators (MMP2, MMP3, and TSLP), the prevalence of circadian oscillations was reduced in those ICU patients with reduced detection (*P* < 0.05, χ^2^ test) (Figure 2, A and B). The prevalence of circadian oscillations also appeared reduced for the remaining 2 inflammatory mediators; however, this was not statistically significant (Figure 2A).

### Reduced detection of circadian clock-gene oscillations is associated with higher inflammatory mediator and C-reactive protein expression.

The association between inflammatory mediator expression and molecular oscillator circadian oscillations was then examined, as this has been previously reported in animal models (12). ICU patients with reduced detection of circadian oscillations had significantly higher inflammatory mediator protein expression for 17 out of 37 measured inflammatory mediators (Figure 2C). Furthermore, when inflammatory mediator protein expression was correlated with the number of oscillatory genes per patient, significant correlations were observed for 19 out of 37 measured inflammatory mediators (*r^2^* = 0.33–0.56, Supplemental Figure 3).

Pentraxin-3, an acute-phase protein, had the greatest fold change of all the measured inflammatory mediators among ICU patient groups. However, this acute-phase protein is not routinely measured in the ICU. Therefore, the expression of an acute-phase protein that is routinely measured (C-reactive protein [CRP]) was also investigated. CRP was 4-fold higher in patients in whom the circadian oscillations in the molecular oscillator were reduced compared with clinical populations in whom the detection of circadian oscillations was normal (ICU or kidney-transplant recipients) (*P* < 0.01, ANOVA, post hoc Tukey’s test; Figure 2D). Similar differences in CRP expression were observed when a threshold of 2 or 4 clock genes was used to stratify normal or reduced circadian rhythmicity (Supplemental Figure 4). CRP expression was also negatively correlated with the number of detected circadian oscillatory clock genes per patient in the ICU in a manner similar to that of pentraxin-3 (*r^2^* = 0.588, Pearson’s; Figure 2E).

### The amplitude of the molecular oscillator is increased following kidney transplantation.

ClinCirc’s ability to define the characteristics of detected circadian oscillations is a key advantage over L-SP, enabling the characterization of detected circadian waveforms. An investigation into the effect of period constraints when added to the final cosinor fit had a very modest effect on amplitude (±5%) and also phase (±0.2 hours) estimation (Supplemental Figure 5) compared with an unconstrained fit in kidney-transplant recipients. Therefore, the period was constrained to be between 18 hours and 30 hours to prevent inappropriate fitting in borderline cases (Supplemental Methods). Since ClinCirc detected at least 3 circadian oscillations per patient after kidney transplantation (Figure 1F), a level of detection seen in 95% of healthy volunteers, the characteristics of these oscillations were investigated further.

**Figure 3:**
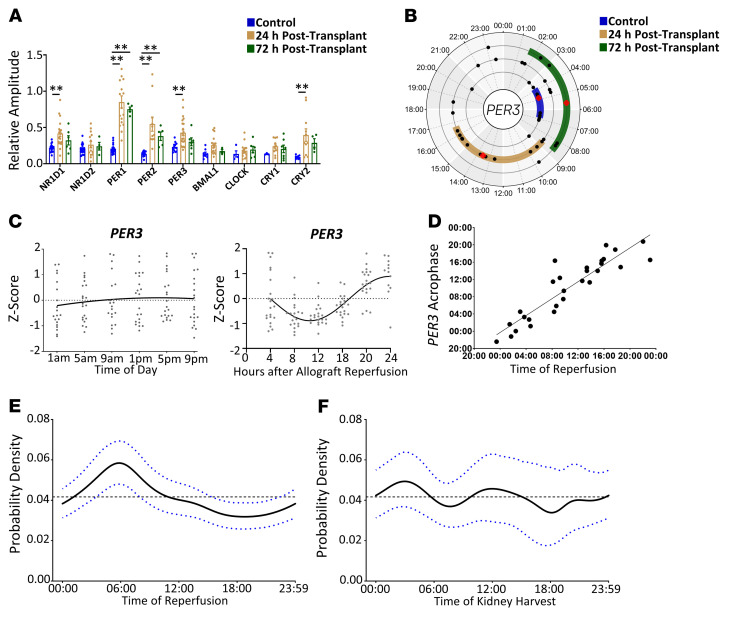
Kidney transplantation induces a phase shift that is associated with altered clinical outcomes. (**A**) The relative amplitude of each clock gene’s circadian oscillation 0 to 24 hours after transplantation (24 hours, *n* = 22) and 48 to 72 hours (72 hours, *n* = 7) after kidney transplantation was compared with the amplitude from healthy volunteers. Data are represented as mean ± SEM. **q < 0.01, 2-way ANOVA, post hoc Dunnett’s. Each circle indicates patient. (**B**) Acrophase plot of PER3’s circadian oscillation 0 to 24 hours and 48 to 72 hours after kidney transplantation was compared with that of healthy volunteers. Circles indicate individual patients. Data are represented as median ± IQR (color band). (**C**) PER3 transcript expression plotted against either time of day or time after allograft reperfusion for the first 24 hours following transplantation (*n* = 22). Circles indicate individual patients. Regression line is shown. (**D**) The acrophase of PER3’s circadian oscillation was compared against time of organ reperfusion immediately after kidney transplantation (24 hours). Circles indicate individual patients. *r^2^* = 0.87 linear regression. (**E**) The prevalence of DGF after kidney transplantation from brain-dead donors was calculated in a 10-year retrospective cohort (*n* = 536). The probability density for DGF was then plotted against allograft reperfusion time. Data are represented as mean ± 95%CI. Gaussian smoothing with bootstrap. (**F**) The probability density of DGF was also plotted against allograft harvest time from the donor. Data are represented as mean ± 95%CI. Gaussian smoothing with bootstrap. For circadian rhythm analysis, data were only plotted if a circadian rhythm was detected. Black dotted lines show uniform lines.

Twenty-four hours after kidney transplantation, the amplitude of circadian oscillations was increased compared with that of healthy volunteers for NR1D1, PER1, PER2, PER3, and CRY2 (*P* < 0.01, 2-way ANOVA post hoc Dunnett’s; Figure 3A). These changes persisted for a minimum of 72 hours following transplantation for both PER1 and PER2 (*P* = 0.01–0.02, 2-way ANOVA post hoc Dunnett’s; Figure 3A).

### The phase of the molecular oscillator is entrained to time of organ reperfusion immediately after kidney transplantation.

The phase of the molecular oscillator following kidney transplantation was then analyzed using ClinCirc. Immediately after transplantation (24 hours), the mean phase for 4 clock genes (NR1D1, NR1D2, PER1, and PER3) was significantly shifted compared with that of healthy volunteers or kidney-transplant recipients 72 hours after kidney transplantation (Figure 3B and Supplemental Figure 6). In contrast, the phase for PER2 was not significantly shifted (Supplemental Figure 6) compared with that of healthy volunteers. Mean expression for PER3 in the cohort did not vary according to time of day (Figure 3C), despite this being reported for healthy volunteers (24) and PER3 having a circadian oscillation in the majority of transplant recipients (Figure 1F) immediately after transplantation.

Therefore, we investigated to determine whether PER3’s circadian oscillation was associated with a different factor. During transplantation, the time at which the donor kidney is reperfused by the recipient’s blood is termed the allograft reperfusion time. This time has been linked to adverse outcomes during transplantation (25). A sinusoidal oscillation was observed for mean PER3 expression when the expression levels were plotted against time after allograft reperfusion (*r^2^* = 0.9, *P* < 0.01; Figure 3C). The strong correlation of PER3’s acrophase with allograft reperfusion time further confirmed this association (*r^2^* = 0.9, *P* < 0.01;Figure 3D). Similar linear correlations were observed for the remaining 8 clock genes’ acrophases, suggesting that the molecular oscillator had been entrained to allograft reperfusion time (*r^2^* = 0.51–0.83, *P* < 0.01; Supplemental Figure 7)

### Delayed graft function, a kidney-transplant complication, oscillates in a circadian manner.

To determine whether this reentrainment of circadian oscillations was associated with adverse outcomes, the incidence of a postoperative complication (delayed graft function [DGF] ref. 26) after transplantation was examined in a 10-year retrospective cohort of kidney-transplant recipients (*n* = 726). Since the incidence of DGF is affected by the donor, the cohort was split according to whether the donor had experienced brain (*n* = 536, DBD) or circulatory (*n* = 190, DCD) death. The probability of DGF from DCD donors did not show a relationship with organ-reperfusion or organ-harvest time (Supplemental Figure 8, A and B). In contrast, DGF in transplanted kidneys from DBD donors had a sinusoidal relationship with allograft-reperfusion time. The probability density doubled for DGF if allografts were reperfused between 4 and 8 am (Figure 3E), with no overlap in 95% CIs between the peak and trough values. No relationship was seen if the probability density for DGF was plotted against organ-harvest time (Figure 3F).

### Comparison of ClinCirc with other mathematical techniques on clinical data.

After comparing ClinCirc with other mathematical methods on simulated data (Figure 1, B and C) and healthy volunteers (Figure 1D), the performance of ClinCirc was compared with other mathematical methods using both of the clinical data sets described above. Two other methods (cosinor and GPR [Matérn]) detected more than 2 oscillatory clock genes per patient in both cohorts and were able to characterize the properties of the waveform, permitting further analysis. In a manner similar to ClinCirc, these methods identified some clock genes as associated with allograft reperfusion time after transplantation (Table 1). In contrast with the ICU cohort, only ClinCirc analysis of circadian rhythmicity revealed an association with CRP, although this did approach significance with the GPR (Matérn) method (*P* = 0.06) (Table 1).

The number of waveforms that each method (ClinCirc, cosinor, and GPR [Matérn]) defined as circadian for the whole clinical cohort was then compared (Supplemental Figure 9A). ClinCirc detected the lowest number of circadian waveforms (*n* = 43), with cosinor detecting 54 waveforms and GPR (Matérn) detecting 63 circadian waveforms. The goodness of fit (*r^2^*) and leverage distance (distance between maximum outlier and predicted oscillation) were then compared between methods. Initially, the performance of ClinCirc was compared with GPR (Matérn) using waveforms that were only described as circadian by one method and not the other. The adjusted *r^2^* was higher for waveforms that ClinCirc deemed circadian but the GPR (Matérn) method did not (Supplemental Figure 9B). In contrast, there was no difference in leverage distance between analysis methods (Supplemental Figure 9C). Then the same method was applied comparing the performance of ClinCirc with cosinor analysis. In this situation, the adjusted *r^2^* was higher for waveforms that ClinCirc deemed circadian but cosinor did not (Supplemental Figure 9D) and the leverage distance was also lower for these waveforms (Supplemental Figure 9E).

## Discussion

ClinCirc analysis of the peripheral oscillator revealed that inflammation is associated with reduced rhythmicity in ICU patients. In the context of kidney transplantation, in which inflammation is suppressed by medication, ClinCirc revealed that the molecular oscillator was shifted and associated with allograft reperfusion time. Although two other methods (cosinor, GPR [Matérn]) also revealed similar findings in kidney-transplant patients, the strongest association was observed with ClinCirc. In the ICU cohort, ClinCirc was the only method out of the 10 tested to reveal an association between rhythmicity and inflammation that has previously been reported in animal models (10–12). One possible explanation for our finding is that we chose our method to fit the underlying biological hypothesis, i.e., that inflammation is associated with circadian disruption. This was not the case, supported by the fact that ClinCirc had comparable performance to existing methods on simulated data as well as in healthy volunteers. Furthermore, ClinCirc’s analysis of waveforms was more accurate than existing methods on PER2:luc data and on the clinical data (higher *r^2^*). Therefore, ClinCirc analysis of circadian oscillations in the molecular oscillator provides translationally important insights into circadian biology in clinical situations in which it is difficult to detect circadian oscillations.

Measuring circadian oscillations in patients is challenging, due to practical considerations that limit the length of the sampling period as well as the number of samples that can be collected. Our data would suggest that the widely used cosinor approach is prone to overfitting in this scenario, which was demonstrated by an increased leverage distance compared with ClinCirc alongside a lower *r^2^* value. The resulting poor specificity could explain why this and previous studies have not observed an association between inflammation and circadian oscillations (27) using this approach. An alternative approach to cosinor analysis is GPR, in which the circadian waveform is defined by a particular covariance kernel. The performance of 2 kernels was evaluated in this study, with the Matérn (28) kernel uncovering clinical associations in the context of kidney transplantation and showing a trend toward an association with inflammation in the ICU (P = 0.06). In a manner similar to what occurred with cosinor analysis, the *r^2^* values of the fit for the GPR (Matérn) method were lower than for ClinCirc, again suggesting that the lack of specificity could hide clinically important associations. Further optimization of kernels is likely to improve this performance; however, GPR techniques are also quite computationally intensive and so would require more resources than ClinCirc to run.

Circadian biology has used multiple mathematical techniques in the past to establish the presence of circadian oscillations, and it is common to pass data through a series of hypothesis tests to address different questions about the oscillations. When testing for the presence of circadian rhythms, the existing techniques tend to run multiple methods concurrently and select the optimal method either via *P* value, e.g., using CirCaN (21), or via a voting strategy, e.g., with MetaCycle (22). In the latter case, results from different techniques can be integrated for *P* values and parameter estimates. Many of these methods were developed for cases in which there are large amounts of data, i.e., frequent samples per period over many periods. Given the sparse data available from the clinical measurements, we adopted a different approach in which the L-SP was used as a preprocessing step to exclude clearly noncircadian rhythms. This mathematical approach is somewhat equivalent to eyeballing a trace to see whether a circadian rhythm is likely. Once its circadian nature is established, the characteristics of the circadian waveform are then defined using cosinor analysis. We opted to constrain the period in this analysis to between 18 and 30 hours, with the minimum being based on the average period of the signal components that can be detected by the L-SP for the sparsest data. These constraints could be optimized further in future experiments with larger data sets. Reassuringly, however, the presence or absence of constraints did not affect the biological associations in the present study.

The association of inflammation with circadian disruption has not been widely reported in human studies, despite previous reports in animal models (29–31) and the identification of RELA as binding to the E-box element, a core transcription binding site in circadian biology (12). This could explain why sepsis has been associated with a lack of circadian rhythmicity in other ICU studies (14, 27, 32–35). The results of our study indicate that decreased circadian rhythmicity is also associated with nonseptic inflammation, suggesting that this phenomenon is more prevalent than previously suspected. In our cohort, almost half (45%) of ICU patients were affected, confirming the high prevalence of this type of circadian dysfunction on ICU patients. This could be one reason for the recently reported poor performance (14) in critical care of a dual-sampling (23, 36) method to ascertain the time of the molecular oscillator. One of the strongest associations with the loss of circadian oscillations was with CRP, a routinely measured acute-phase protein. Therefore, if other studies confirm our findings, CRP could be used as a biomarker for circadian rhythmicity, potentially improving the accuracy of single or dual time-stamping techniques.

A key strength of ClinCirc is the characterization of a detected circadian oscillation. In kidney-transplant recipients, a gain in rhythmicity was observed, despite major operations being recognized as drivers of inflammation (37). This, however, is not the case after kidney transplantation, since recipients are given immunosuppressive therapy, which suppresses inflammation, causing the mean CRP to be 14.9 mg/L in our cohort (Supplemental Table 3). Glucocorticoids are among the types of immunosuppressive medications administered in our program. This medication has previously been reported to enhance the amplitude of circadian rhythmicity through direct modulation of the molecular oscillator (38), resulting in a phase shift in its oscillation (38). In our program, glucocorticoids are given around the time of allograft reperfusion, potentially explaining the observed phase shift in the molecular oscillator. These phase shifts have adverse consequences in a number of studies (9) and therefore could explain why renal transplant outcomes show diurnal variation at one year (25) and why, in another study, DGF oscillated in a diurnal manner (39). Our study now extends this time-of-day variation for DGF, showing that this variation is circadian. It should be noted, however, that we found DGF incidence higher in the morning, in line with our previous report after lung transplantation (40), whereas the previous study in kidney transplantation found the prevalence was higher in the evening (39). This apparent discrepancy could be explained by differences in the administration of glucocorticoids in the different programs, as this is known to vary in renal transplantation, or the methods (diurnal versus circadian) used to analyze the data. Furthermore, the underlying pathology, ischemia-reperfusion injury, has previously been reported to be under circadian control in other clinical scenarios (3, 40–42).

When interpreting the results, some limitations should be considered. The first is that this study, in line with other observational studies, can only determine associations rather than causation. The second is the modest size of the clinical cohorts. Because of this, we did not have the power to run a multivariate analysis, which could control for confounders in the study. To minimize the effect of this, we set strict recruitment criteria for ICU patients. This could explain why the only significant differences between groups with normal and reduced circadian rhythmicity was CRP (Figure 2D and Supplemental Table 4). The strict recruitment criteria, however, do limit the generalizability of the results when applied to an unselected group of ICU patients. In this study, we did not measure melatonin, which several studies use to correct circadian phase (23, 24). This is because in the hospital care setting, melatonin is affected by surgery (43) and also light (44), potentially making it an unreliable marker of circadian phase. Care must also be taken in interpreting the signals that ClinCirc deems to be circadian. Passing the L-SP preprocessing stage means simply that the 24-hour signal has the highest amplitude. If the higher harmonics have comparable amplitudes, then the signal will not be well approximated by a single sinusoidal wave with a period of approximately 24 hours. Thus, a “circadian” signal reported by ClinCirc will not necessarily look like a “perfect” archetypal circadian oscillation, but the evidence presented in this paper suggests that such signals contain meaningful biological information.

In conclusion, ClinCirc is a robust method that permits characterization of circadian changes from clinical blood samples collected over a 24- to 48-hour period. In this study, it is revealed that inflammation is associated with loss of circadian rhythmicity in the ICU and that a kidney-transplant recipients’ clock is entrained to the time of operation. This shift in phase potentially explains the circadian regulation of DGF, and therefore, the use of ClinCirc in other studies is likely to provide insights into the translation of circadian mechanisms.

## Methods

### Mathematical analysis.

Ten mathematical techniques, including the L-SP, least-squares cosine fitting, and GPR, were used to analyze circadian oscillations and are described fully in Supplemental Methods. The mathematical technique ClinCirc was created, in which the presence or absence of a circadian rhythm is determined by a L-SP test combined with a cosine fit with a loose period filter. A constrained cosinor analysis was then used to estimate phase and amplitude. Specifically, the period was constrained between 18 and 30 hours based on the L-SP harmonic and the amplitude was constrained to (2 SD of mean), i.e., a 95% CI. A full description of ClinCirc can be found in Supplemental Methods, and the code to run it can be obtained from figshare.

### Study patients.

ICU patients who received invasive ventilation (tracheostomy or endotracheal tube) and were likely to survive 48 hours, as identified by the admitting ICU physician, were considered for entry between September 2017 and September 2019 if they were aged over 18. Patients were excluded a priori if they had BMIs over 35, nonreversible organ dysfunction, psychiatric illness requiring inpatient treatment within the last 12 months, a history of shift work, a medically treated sleep disorder (including use of continuous positive airway pressure [CPAP]), end of life care, alcohol intake greater than 70 units per week in last year, nonreversible end-stage renal failure, a cardiac arrest in the last year, cancer treatment or surgery in the last 30 days, suspicion of a highly transmissible infection, were pregnant or breastfeeding, or had a permanent neurological deficit due to the potential effects this could have on circadian oscillations. Inclusion and exclusion criteria were set a priori based on discussion between the investigators and a review of the literature. Diagnosis and clinical characteristics of the patients were independently verified by review of the charts by two General Medical Council–registered doctors and are listed in the patient demographics. The number of cases during the sampling period determined the sample size. The sample size used to evaluate alterations to the peripheral oscillator was comparable to those in other studies with similar objectives (14, 27, 32–35). The sample size that evaluated DGF was limited by a change in transplant protocol in the unit in 2007 and is comparable to that in our previous study reporting circadian oscillation in primary graft dysfunction after lung transplantation (40). Blood samples were taken for a 48-hour period within 24 hours of consent into the study.

Patients undergoing cadaveric kidney transplantation at Manchester Foundation NHS trust between 2015 and 2019 were considered for recruitment. Consent was provided before transplantation when the retrieval team was retrieving the organ. Following consent, blood was taken every 4 hours for the first 24 hours after the operation (24 hours) or between 48 and 72 hours after the operation (72 hours). Patients were excluded from the study if the surgeon thought they had a high probability of needing hemodialysis before or during the circadian sampling period. Clinical parameters, e.g., CRP, were recorded from the clinical chart over the sampling period.

The data for DGF were taken from a 10-year retrospective service evaluation starting when the transplant protocols were modified in 2007 at MFT. Patients were identified following analysis of the transplant center’s database. The presence of delayed graft failure was then identified following review of the patient’s health-care record. DGF was defined for this study as the need for dialysis within the first 7 days after transplantation. The time of organ harvest and organ reperfusion as well as the type of cadaveric transplant were also obtained from the patient’s health-care record.

### RNA sample collection and qPCR.

Blood (3 ml) was collected in Tempus Blood RNA tubes for RNA analysis. Each tube was inverted 3 times, lysing the blood, and then the tube was frozen (–20°C) until analyzed. RNA was then extracted from the sample using Tempus Spin RNA Isolation Reagents following the manufacturer’s instructions and subsequently converted into cDNA using an Applied Biosystems High Capacity RNA-to-cDNA kit. Quantitative PCR (qPCR) was performed using prevalidated PrimePCR TaqMan primer assays from Bio-Rad, using TBP as the housekeeping gene.

### Inflammatory mediator analysis.

Blood (6 ml) was drawn into Vacutainer Lithium Heparin Tubes (BD). Following centrifugation at 1,500*g* for 10 minutes, the plasma was aliquoted and stored at -80°C until further analysis. The protein concentration of inflammatory mediators was analyzed using the Pro Human Inflammation 37-plex Bio-Plex per the manufacturer’s instructions (Bio-Rad).

### Human volunteer data.

Two studies were analyzed for control data. For the first study (23), the data were downloaded from the NCBI’s Gene Expression Omnibus database (GEO GSE39445). For every healthy volunteer studied under control conditions, the probe with the highest expression for a specific gene was analyzed using ClinCirc. Samples were taken from whole blood as outlined in the original study method, and this was used as the control patient set unless otherwise specified. For the second study (23), the RNA-Seq profile of monocytes in the peripheral blood was analyzed using ClinCirc according to the time the blood sample was taken.

### Code.

The ClinCirc algorithm is available at https://figshare.com/s/700cedb00253847212ae along with instructions, which are available at https://figshare.com/s/d492ca6f244380f8e650 An example data set can be found at https://figshare.com/s/7a8d545272046bf53f22

### Statistics.

Statistical analysis was performed using either GraphPad Prism, version 9 (ANOVA and post hoc Tukey’s or Dunnett’s test, *t* tests, χ^2^ test) or Mathematica (Watson *U^2^* test) to compare phases. GPR (periodic) was implemented using custom routines written in MATLAB. GPR (Matérn) was implemented using previously published routines (28) written in Python. One blood sample was not taken for 1 of the ICU patients and for 2 of the kidney-transplant recipients; however, ClinCirc could still be run, since the method adjusts for this. A *P* value of less than 0.05 was considered significant.

### Study approval.

This clinical study was approved by 2 research ethics committees (REC references 17/NW/0030 and 15/NW/0338) as well as the institution (Manchester University NHS Foundation Trust). Written, informed consent was obtained prior to patient enrollment. Consent was provided by patients if they were deemed to have capacity; otherwise, assent was provided by a professional or close relative, which was subsequently confirmed by the patient when they regained capacity.

## Author contributions

JFB, GK, and PSC conceived the study, analyzed the data, and wrote the manuscript. CJ, TH, OEA, and ALH developed and applied the mathematical methods and helped write the manuscript. SP and TS helped acquire data and critically appraised the manuscript. DVD, JG, TF, AW, JBS, MKR, TA, and PD all helped with the design of the study and study analysis and provided critical feedback on the manuscript.

## Supplementary Material

Supplemental data

Trial reporting checklists

ICMJE disclosure forms

## Figures and Tables

**Table 1:**
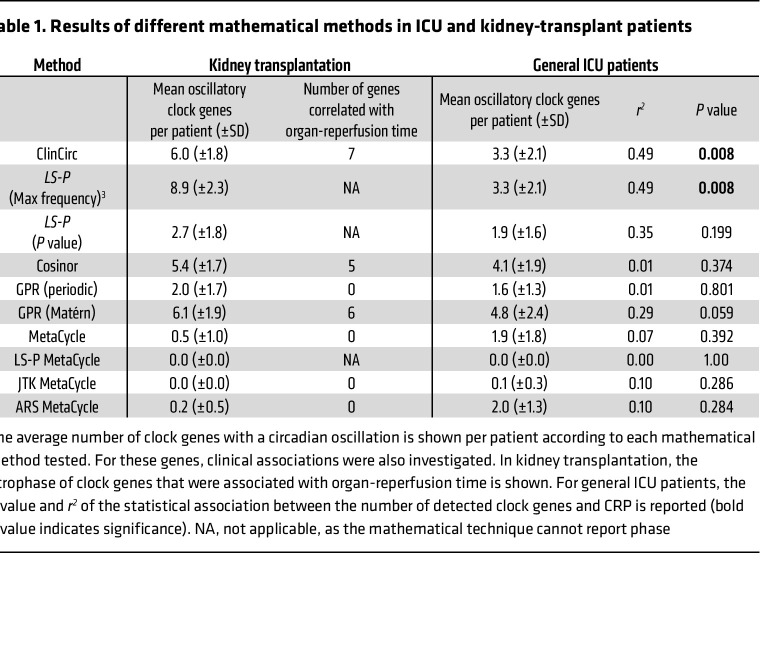
Results of different mathematical methods in ICU and kidney-transplant patients

## Version 1 12/20/2022

In-Press Preview

## Version 2 02/15/2023

Electronic publication
