# Supplementary material for: ClinCirc identifies alterations of the circadian peripheral oscillator in critical care patients
Source: J Clin Invest. 2023 Feb 15;133(4):e162775. doi: 10.1172/JCI162775 (PMC9927929; doi:10.1172/JCI162775)
Supplement: Supplemental data [file jci-133-162775-s104.pdf]

Supplementary Figures and Tables

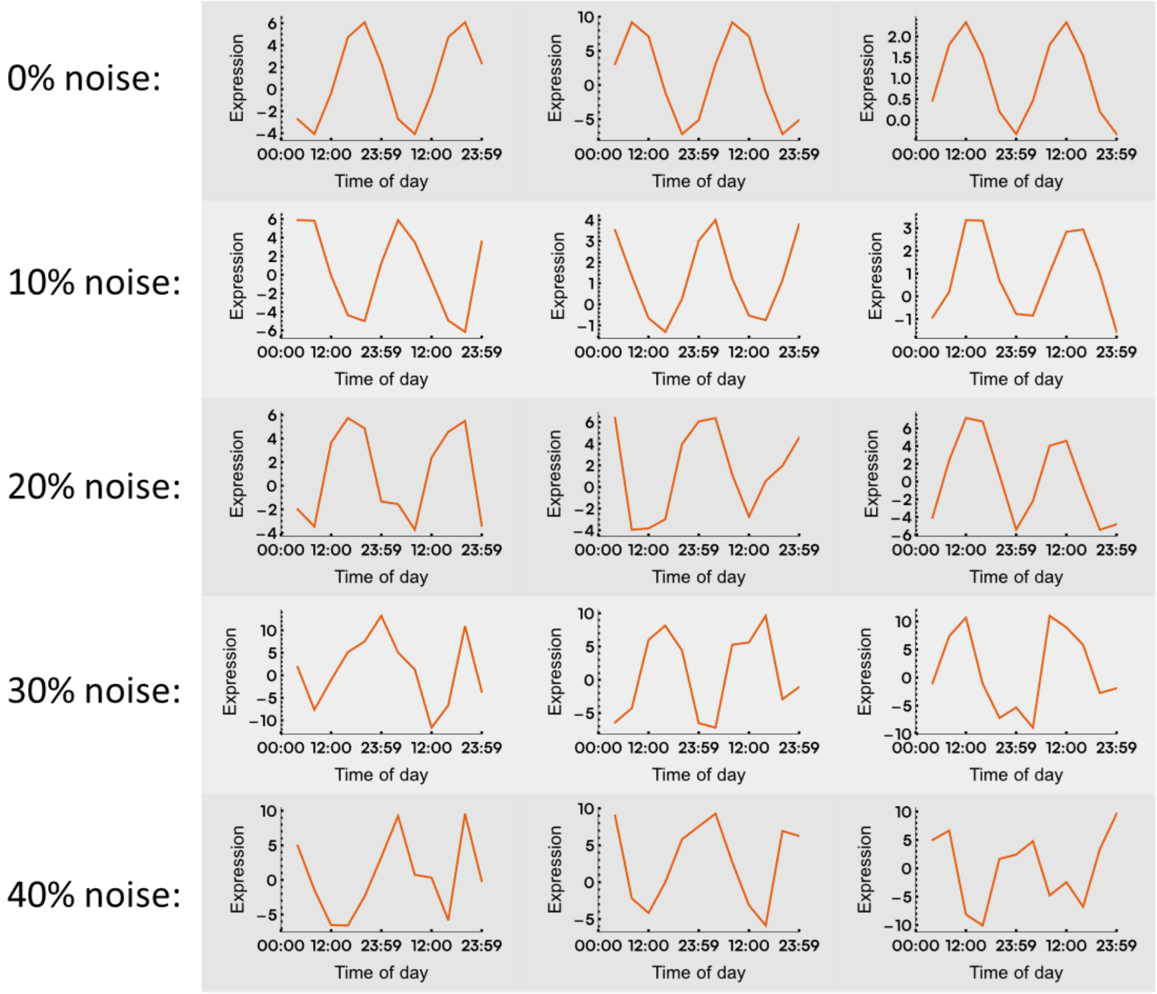

Supplemental Figure 1: Visualisation of noise that was added to sinusoidal oscillations to estimate sensitivity: Different noise levels were added to the synthetic data based on the % amplitude of the original wave. Noise was added simultaneously to the amplitude, period, and phase of the wave each time a point was sampled. To visualise this, 3 noisy oscillations were simulated with data points being shown. **-** =Lines from exemplar signals.

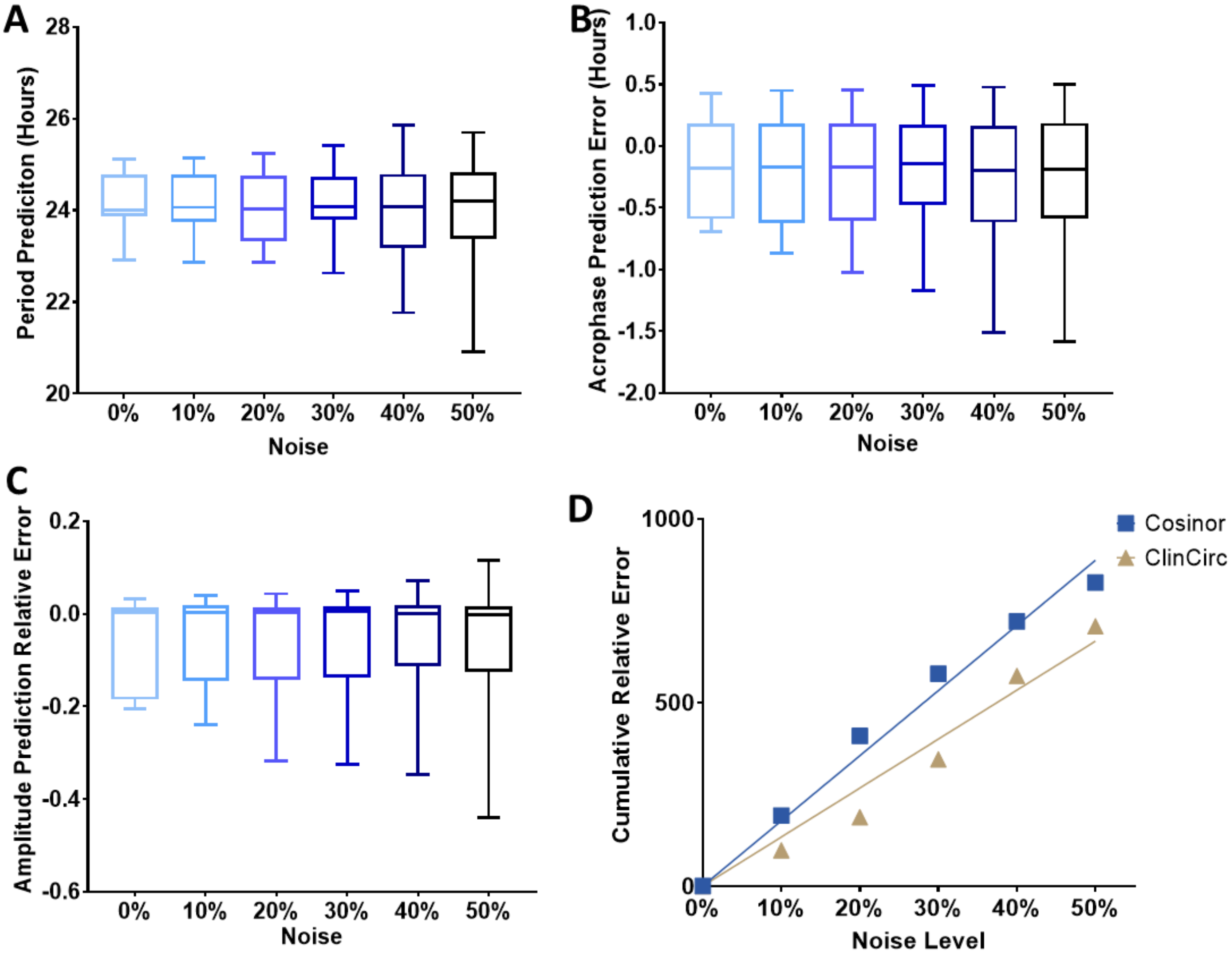

Supplemental Figure 2: The effect of artificial noise on ClinCirc accuracy assessment of PER2::luc bioluminescence data: Bioluminescence from cultured lung tissue in PER2::luc mice was analysed using ClinCirc at a 4-hour sampling frequency and then compared to the original data sampled at a 1-minute frequency. Different noise levels (0-40%) were then added to the data. **(A)** Period **(B)** Acrophase and **(C)** Amplitude was then calculated using ClinCirc (*n*=250; Box=median ± interquartile range; whiskers denote maximum and minimum). **(D)** The cumulative relative error combining the errors for period, acrophase and amplitude using two different mathematical tests (ClinCirc and Cosinor) after the addition of six different levels of noise (0-50%, *n*=250 simulations). A linear regression line is shown.

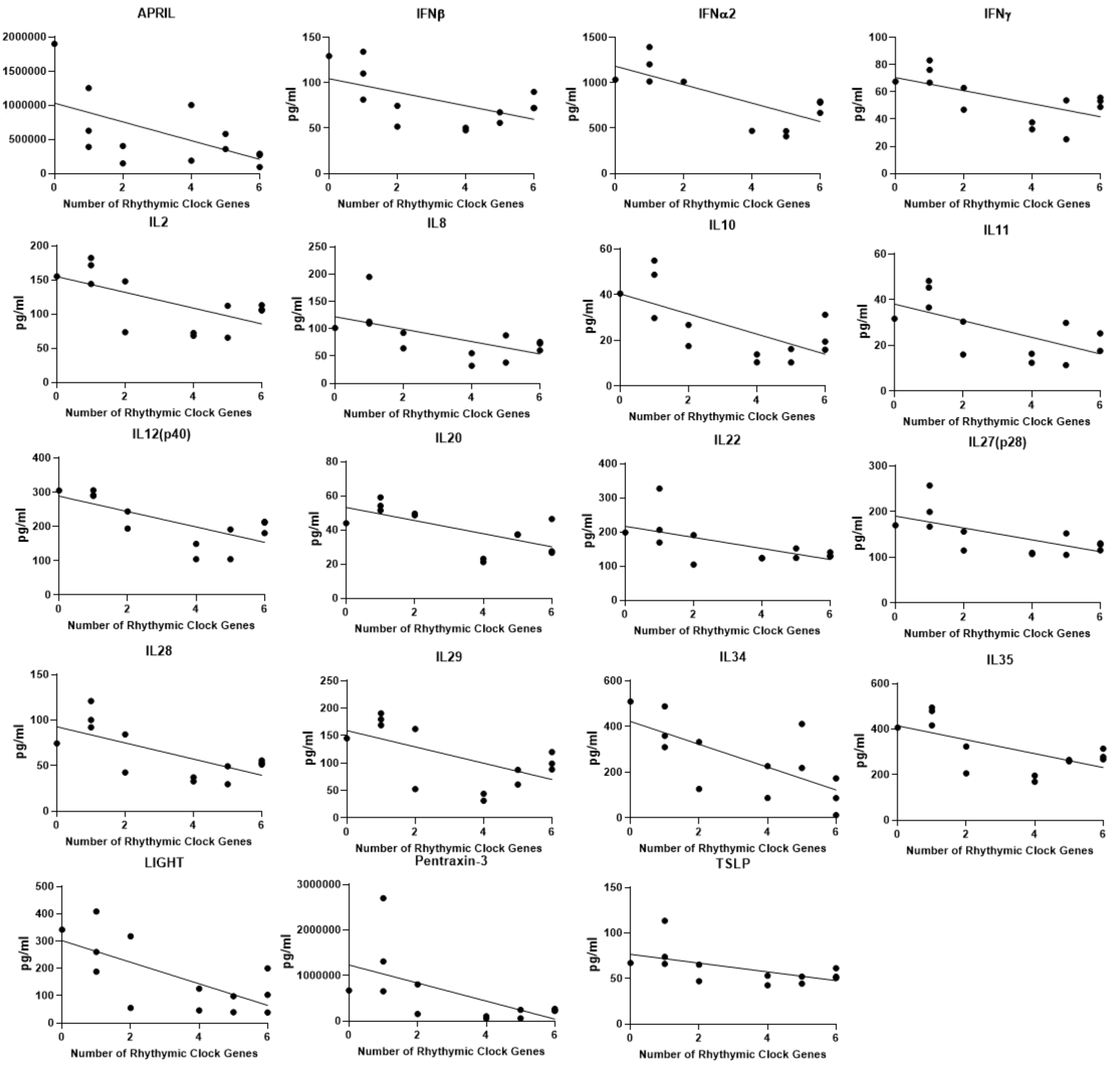

Supplemental Figure 3: The correlation of inflammatory mediator with the number of rhythmic clock genes identified by ClinCirc: The expression of 37 inflammatory plasma mediators were compared to the number of genes in the molecular oscillator that had a circadian rhythm defined by ClinCirc. Significant correlations are shown for each of the nineteen inflammatory mediators where this was observed.

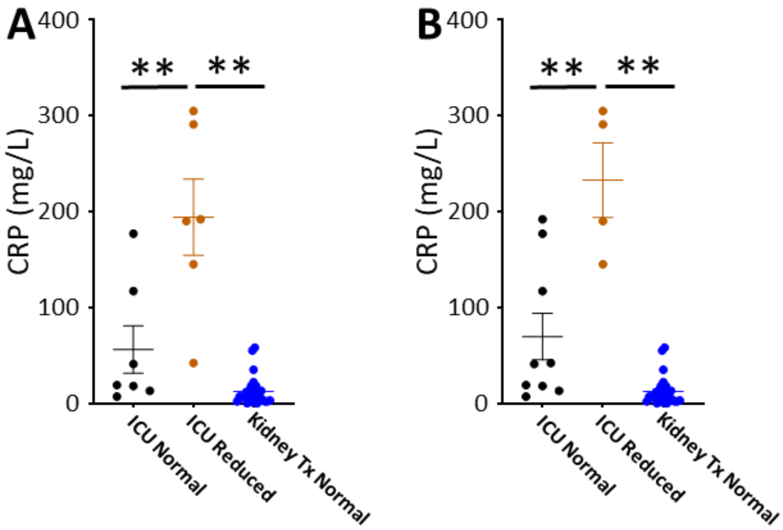

Supplemental Figure 4: Effect of Different thresholds to determine circadian rhythmicity The effect of different thresholds on circadian rhythmicity was explored for the association with CRP in ICU patients (A) Reduced was defined as the presence of 4 or less clock genes in an ICU patient which had a circadian oscillation (B) Reduced was defined as the presence of 2 or less clock genes in an ICU patient which had a circadian oscillation (**=p<0.01; ANOVA post-hoc Tukey)

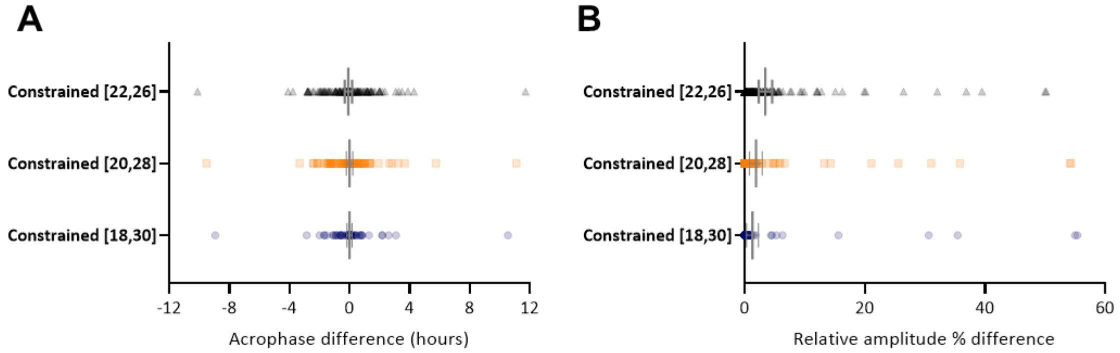

Supplemental Figure 5: The effect of changing the constraints for Cosinor analysis in a cohort of kidney transplant recipients: After pre-processing the effect of adding three different constraints to the period fit in the final cosinor fit was investigated. The period was constrained to be between 22-26h, 20-28h or18-30h. The effect of this on **(A)** acrophase and **(B)** amplitude measurement is shown compared to an unconstrained cosinor fit. Mean±95% confidence intervals are shown (●= single waveform)

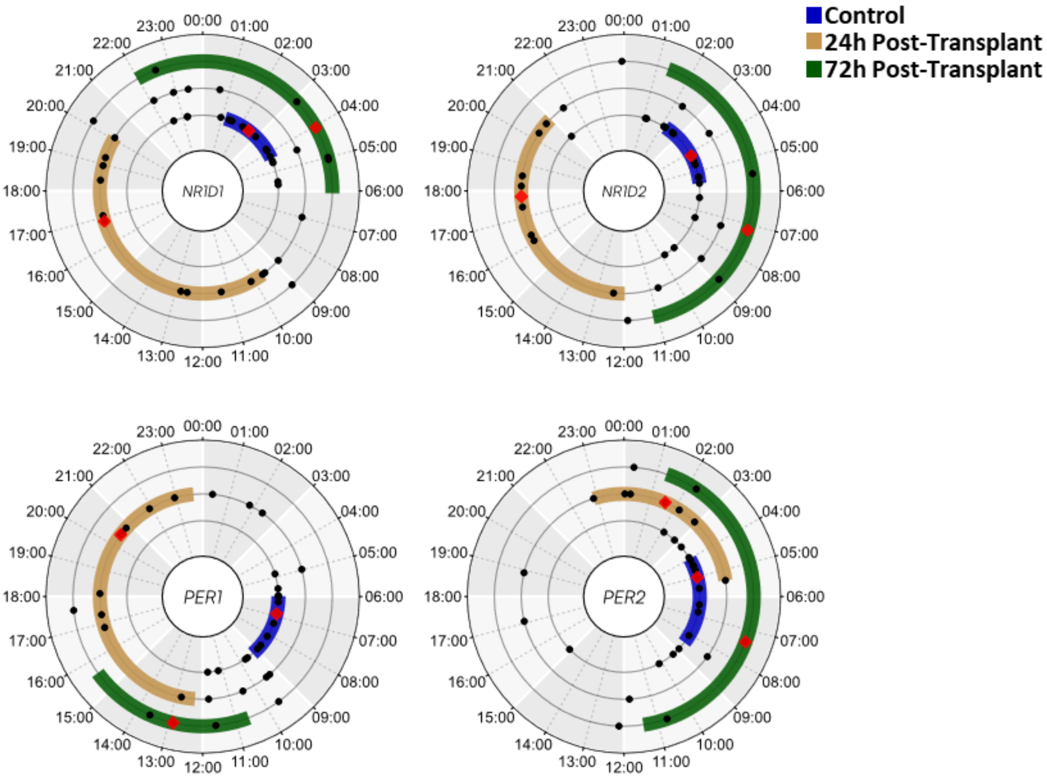

Supplemental Figure 6: Phase plots of the molecular clock following kidney transplantation. Phase plot of the molecular oscillator genes *NR1D1*, *NR1D2*, *PER1*, *PER2* in kidney transplant patients 0-24 hours (*n*=23, 24 hours) and 48-72 hours (*n*=7, 72 hours) after transplantation (●= individual patients, ◆=median±IQR (colour band)). Patients are only shown if they had a detectable circadian oscillation for that gene.

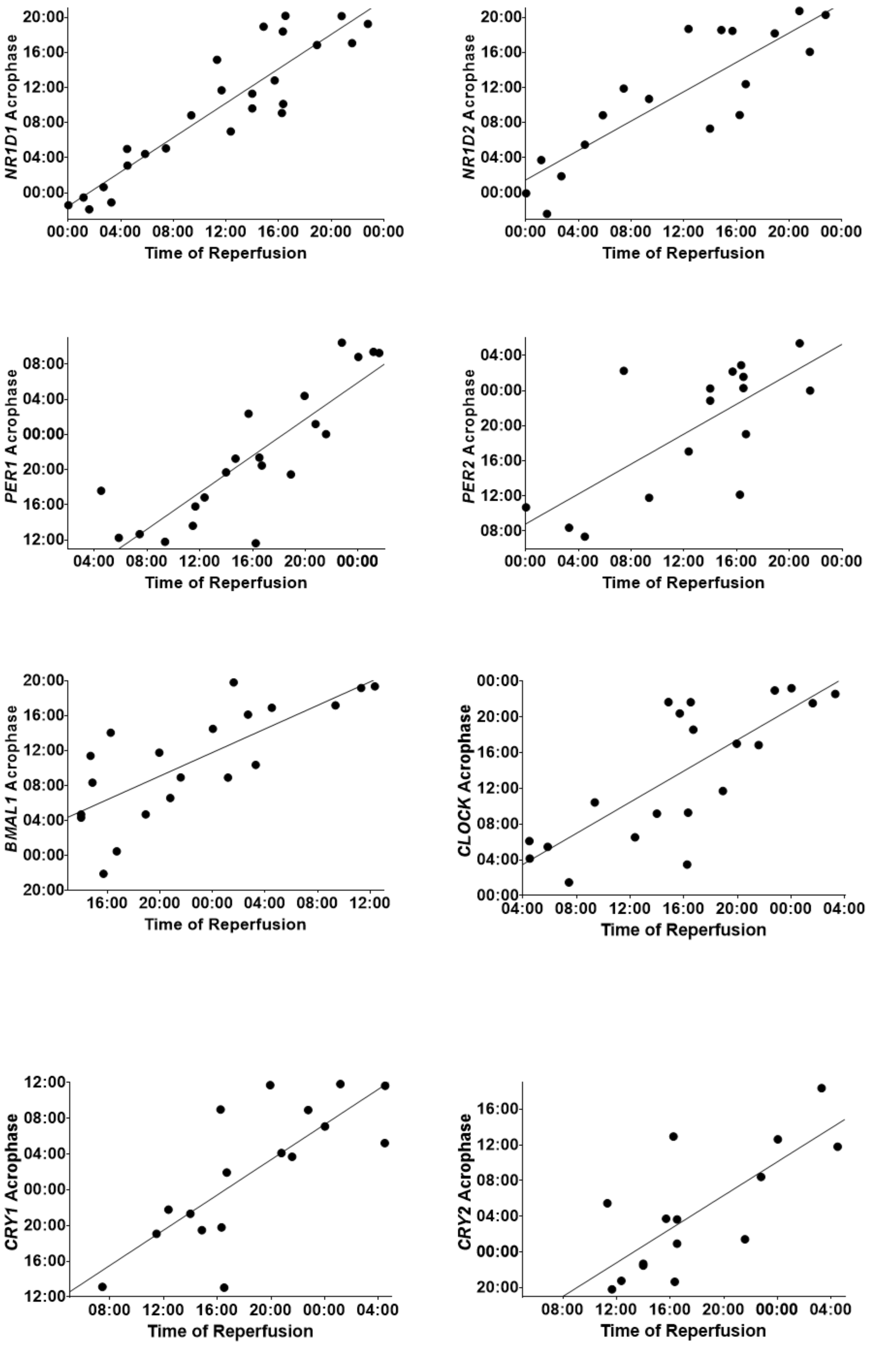

Supplemental Figure 7: Circadian rhythms are shifted according to allograft reperfusion time following kidney transplantation: Molecular oscillator genes were measured in the blood of kidney transplant recipients over the first 24 hours after transplantation. For patients where the clock gene had a circadian oscillation, the acrophase was plotted against time of allograft reperfusion (●= individual patients with a circadian oscillation, a linear regression line is shown r^2=0.51-0.83).

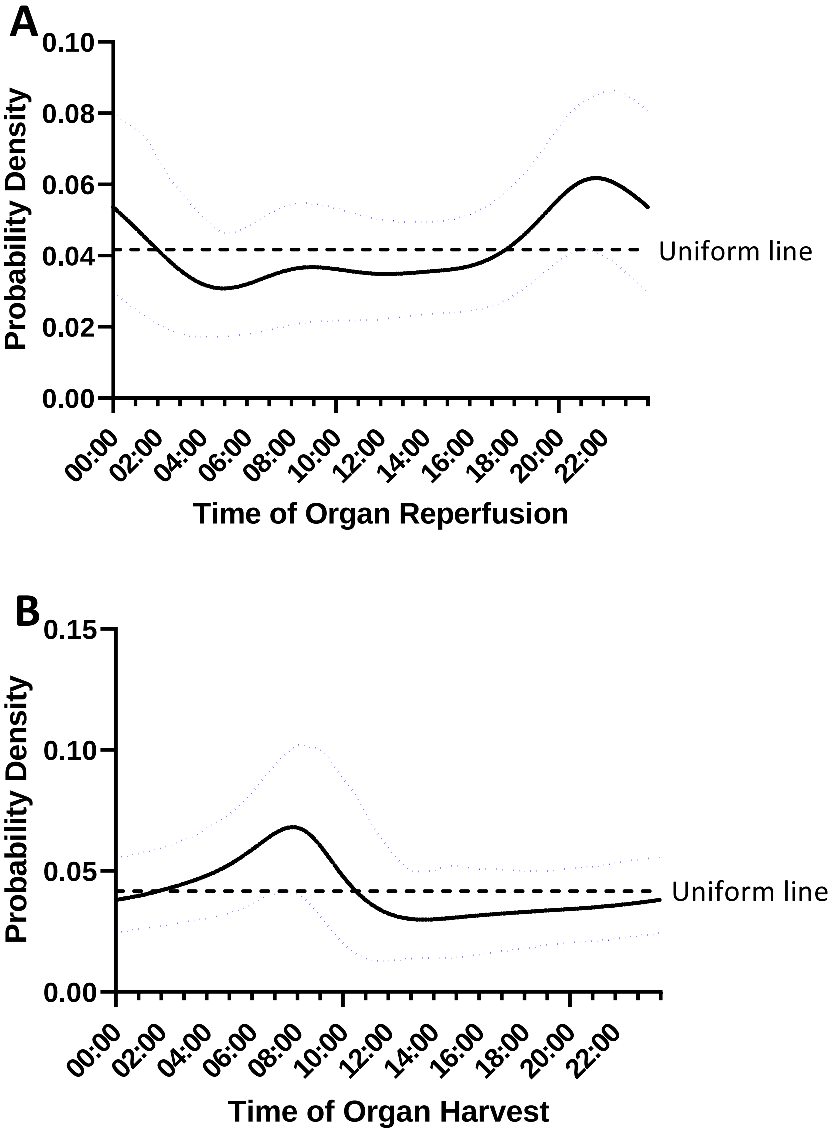

Supplemental Figure 8: Oscillation in delayed graft function and allograft rejection following kidney transplantation with allografts from circulatory dead donors: (A) Probability density of delayed graft function against time of allograft reperfusion utilising kidneys from circulatory dead donors (- =mean probability density ±95%CI, Gaussian smoothing with bootstrap) **(B)** Probability density of delayed graft function against time of organ harvest utilising kidneys from circulatory dead donors (- =mean probability density ±95%CI, Gaussian smoothing with bootstrap).

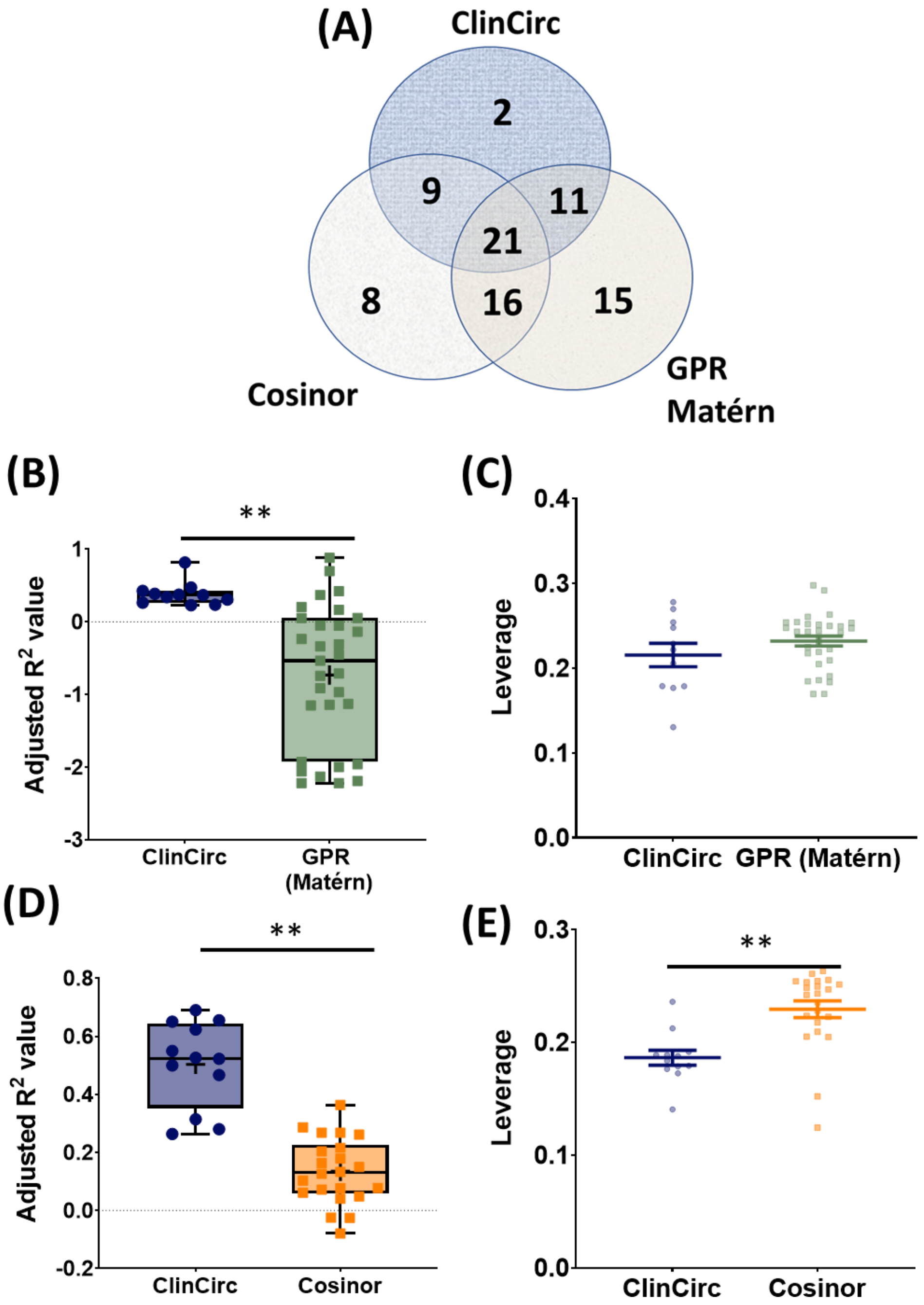

Supplemental Figure 9: Normalised leverage and Adjusted R^2 values for ClinCirc, Cosinor and Gaussian process regression (Matérn): Circadian oscillations for the molecular oscillator genes in the ICU patient cohort were analysed using three mathematical methods. **(A)** Is a Venn diagram depicting the overlap for the three detection methods. The numbers represent how many gene oscillations belong in that section. **(B)** Displays the adjusted R^2 values for the oscillations detected by ClinCirc or GPR Matérn analysis which the other method did not detect. **(C)** Leverage distances were calculated for the oscillations detected by ClinCirc or GPR Matérn which the other method did not detect **(D)** Shows the adjusted R^2 values for the oscillations detected by ClinCirc or Cosinor analysis which the other method did not detect. **(E)** Leverage distances were calculated for the oscillations detected by ClinCirc or Cosinor analysis which the other method did not detect

Mathematical Method	**Sensitivity for a 24-hour sampling period (%)**			**Sensitivity for a 48-hour sampling period (%)**		
	0% Noise	20% Noise	40% Noise	0% Noise	20% Noise	40% Noise
ClinCirc	100	98.65	88.28	100	98.92	76.92
LS-P Frequency	100	98.78	89.76	100	98.92	76.92
LS-P (p-value)	100	83.22	41.42	100	97.12	55.54
Cosinor	100	88.7	66.30	100	98.02	78.94
Gaussian (Periodic)	0	45.6	34.6	0	79.8	38.80
Gaussian (Matérn)	100	88.4	84.00	99.8	92.4	69.60
MetaCycle	100	30.52	0.06	100	95.38	42.74
LS-P MetaCycle	0	0.0	0	100	0.0	0
JTK MetaCycle	100	0.0	0	100	80.9	0.6
ARS MetaCycle	45.62	1.72	0.04	100	95.92	43.02

Supplemental Table 1: Sensitivity of ten mathematical methods in detecting circadian oscillations. A cosinor wave with a period of 24 hours was sampled every 4 hours over 24 or 48 hours. Noise was added to the amplitude, period, and phase of the wave at every data point sampled (*n*=5,000).

		ICU
Age (years)		60.85 (±13.39)
Sex (% male)		62% (n=8)
Admission Diagnosis	Pneumonia	4
	Trauma	3
	Acute Abdomen	5
	Other*	1
Ventilation	FiO_2 (%)	35.50 (±7.17)
	SpO_2 (%)	96.12 (±1.93)
	Peak Inspiratory Pressure (PIP) (cmH_2O)	19.86 (±5.6)
	Peak End Expiratory Pressure (PEEP) (cmH_2O)	7.10 (±2.16)
	Tidal Volume (mls)	424.33 (±121.37)
	Minute Ventilation (L)	9.30 (±3.03)
Blood Gas	pH	7.42 (±0.04)
	pCO_2 (kPa)	5.71 (±1.24)
	pO_2 (kPa)	11.62 (±2.73)
Cardiovascular	Heart Rate (beats/min)	88.57 (±22.81)
	Mean Arterial Pressure (mmHg)	78.65 (±10.92)
	Noradrenaline (mg/Hr)	0.20 (±0.33)
Temperature (°C)		36.68 (±0.79)
GCS		9.62 (±5.16)
Haematology	White Blood Cell count (10^9/L)	13.78 (±8.35)
	Neutrophil count (10^9/L)	10.70 (±6.66)
	Monocyte count (10^9/L)	0.91 (±0.51)
	Lymphocyte count (10^9/L)	1.34 (±0.57)
Biochemistry	Sodium (mmol/L)	139.70 (±4.10)
	Potassium (mmol/L)	4.22 (±0.55)
	Creatinine (µmol/L)	113.00 (±85.01)
	Bicarbonate (mmol/L)	25.00 (±5.12)
SOFA Score		7.08 (±4.97)

Supplemental Table 2: Patient demographics for ICU patients: Variables are expressed as mean (±SD) or as percentages. SOFA score= Sequential Organ Failure Assessment score(1) , FiO_2= Fraction of Inspired Oxygen *Other was suspected PE with reversible renal failure

Age	55.7 ± 13.2 years
Male	15 (52%)
Cold Ischaemic Time	13.7 ± 5.9 (hours)
Warm Ischaemic Time	27 ± 18.5 Minutes
Donor Type	14 Brain Death<br>15 Cardiac Death
Pre-op Creatinine	533±214.8 µmol/L
CRP during circadian measurement	14.9 ± 19 mg/L

Supplemental Table 3: Patient demographics for Kidney transplant recipients: Clinical variables are expressed as mean (±SD) or as numbers/ percentages.

		Normal detection (n=7)	Reduced Detection (n=6)	P Value
Age (years)		62.57 (±13.87)	58.83 (±13.79)	0.637
Sex (% male)		71% (n=5)	50% (n=3)	0.592
Admission Diagnosis	Pneumonia	3	1	n/a
	Trauma	2	1	
	Acute Abdomen	2	3	
	Other*	0	1	
Ventilation	FiO_2 (%)	36.84 (±8.81)	33.93 (±4.97)	0.490
	SpO_2 (%)	95.64 (±2.03)	96.68 (±1.82)	0.357
	Peak Inspiratory Pressure (PIP) (cmH_2O)	18.22 (±5.81)	21.50 (±5.37)	0.334
	Peak End Expiratory Pressure (PEEP) (cmH_2O)	6.20 (±1.53)	8 (±2.45)	0.159
	Tidal Volume (mls)	377.69 (±160.86)	470.98 (±36.2)	0.196
	Minute Ventilation (L)	7.86 (±3.25)	10.73 (±2.17)	0.102
Blood Gas	pH	7.40 (±0.04)	7.43 (±0.04)	0.241
	pCO_2 (kPa)	5.87 (±1.63)	5.51 (±0.66)	0.617
	pO_2 (kPa)	10.49 (±1.93)	12.94 (±3.09)	0.110
Cardiovascular	Heart Rate (beats/min)	82.34 (±19.62)	95.83 (±25.85)	0.308
	Mean Arterial Pressure (mmHg)	81.18 (±12.4)	75.69 (±9.09)	0.390
	Noradrenaline (mg/Hr)	0.95 (±1.84)	4.09 (±5.45)	0.178
Temperature (°C)		36.5 (±0.90)	36.96 (±0.68)	0.464
GCS		10.73 (±5.06)	8.34 (±5.44)	0.429
Haematology	White Blood Cell count (10^9/L)	11.20 (±5.27)	16.78 (±10.68)	0.246
	Neutrophil count (10^9/L)	8.66 (±4.6)	13.08 (±8.27)	0.248
	Monocyte count (10^9/L)	0.87 (±0.41)	0.96 (±0.64)	0.775
	Lymphocyte count (10^9/L)	1.41 (±0.48)	1.27 (±0.71)	0.676
Biochemistry	Sodium (mmol/L)	139.57 (±3.74)	139.83 (±4.83)	0.914
	Potassium (mmol/L)	4.17 (±0.45)	4.28 (±0.69)	0.733
	Creatinine (µmol/L)	108.43 (±96.04)	118.33 (±78.83)	0.844
	Bicarbonate (mmol/L)	26.57 (±5.46)	23.37 (±4.66)	0.300
SOFA Score		5.00 (±3.56)	9.50 (±5.58)	0.106

Supplemental Table 4: Patient demographics for ICU patients categorised by detected circadian rhythmicity: Variables are expressed as mean (±SD) or as percentages (numbers). SOFA score= Sequential Organ Failure Assessment score (1), FiO_2= Fraction of Inspired Oxygen. One "normal detection" patient received oxygen without ventilation and therefore we were not able to obtain data regarding PIP, PEEP, tidal volume or minute ventilation from this patient. *Other was suspected PE with reversible renal failure

References:

1. Vincent JL, Moreno R, Takala J, Willatts S, De Mendonça A, Bruining H, et al. The SOFA (Sepsis-related Organ Failure Assessment) score to describe organ dysfunction/failure. On behalf of the Working Group on Sepsis-Related Problems of the European Society of Intensive Care Medicine. *Intensive Care Med.* 1996;22(7):707-10.

Supplementary Methods

1 Assessing circadian rhythmicity – computational methods

In the following sections, we describe three core methods used in the paper. These are: (i) (Fast) Fourier–Transform based methods, which can determine whether a 24 hour signal is dominant, provided that the sample interval divides 24 hours; (ii) non-linear, least-squares fitting to a sinusoidal waveform, so-called cosinor analysis; and (iii) maximum likelihood, Gaussian process regression (GPR), using a sum of linear and periodic kernels in the covariance function.

2 Fast Fourier Transform (FFT) and Lomb–Scargle Periodogram

Fourier methods are based on a statistic known as the periodogram, $I(f)$, a function of the frequency f which indicates the contribution of that frequency to the overall signal: *e.g.* for a pure sinusoidal signal of frequency F, the periodogram is only non-zero when $f = F$. For uniformly sampled data

$$I(f) = \frac{1}{N}\left|\sum_{n=1}^{N} x_n e^{-2\pi i f t_n}\right|^2, \tag{1}$$

where N is the total number of data points and (t_n, x_n) are the time points and sample values, the periodogram can be efficiently calculated at the harmonic frequencies $f_n = 2\pi n/N$ via the Fast Fourier Transform. If the data are not uniformly sampled then the alternative Lomb–Scargle periodogram is used, which corresponds to finding the periodogram that arises from finding the best–fit sinusoids at each frequency in a least-squares sense (1). We define a signal to be circadian if the maximum value of the periodogram occurs at a frequency corresponding to a period of 24 hours. Fisher's g–statistic is the normalised value of the maximum value of the periodogram, *i.e.* the maximum value divided by the sum of all values at the harmonic frequencies. This statistic has the value 1 if the signal is a pure sinusoid and decreases with increasing components at other frequencies (noise).

When these methods are applied to pure Gaussian noise, peaks will still occur in the periodogram. In order to differentiate between spurious peaks cause by noise and genuine peaks caused by a signal in the data, a false alarm probability (FAP) is employed to quantify significance. For data consisting of pure Gaussian noise, the values of the periodogram follow a χ^2 distribution with 2 degrees of freedom (2). However, this is the distribution of one particular (arbitrarily chosen) frequency rather than the distribution of the largest peak in the periodogram. The distribution of the largest peak is not easy to calculate since the value at each frequency is correlated with the values at other frequencies and so approximations must be applied. To this end, we turn to computational methods and leverage the bootstrap approximation. To bootstrap the periodogram, the temporal coordinates, t_n, remain fixed but the observational values x_n are resampled with replacement. This generates a bootstrap sample $(t_1, x_1^*), \ldots, (t_n, x_N^*)$ where each x_i^* is chosen uniformly at random from the original times series sample

values $\{x_1, \ldots, x_N\}$. The maximum value of the periodogram applied to this resample is computed and then this procedure of resampling and calculation is repeated. For enough resamples, the empirical distribution of such maxima will approximate the same marginal distribution as the original time series (3). The bootstrap approach was used since it produces a robust estimate by making few assumptions about the form of the periodogram distribution.

3 Least-squares fitting (cosinor analysis)

We perform a least-square fit to the function

$$x(t) = m + A\cos\left(\frac{2\pi\lambda}{24}t + \phi\right),$$

by first assuming a period of 24 hours ($\lambda = 1$), in which case the model is linear in the parameters m, $A\cos(\phi)$ and $A\sin(\phi)$ (4). The linear system is solved to find m, which is then subtracted from the signal. We next perform a non-linear least-squares fit to the case when $m = 0$, treating the frequency as an unknown, but using the results from the linear analysis as an initial guess.

After the non-linear fit, if the amplitude $A > 2\sigma_{\text{data}}$, then the non-linear least-squares fit is recalculated, but using a constrained fit to ensure that $A \leq 2\sigma_{\text{data}}$. Once the amplitude has been tested, we next test the period to ensure that it is neither less than 4 hours, nor greater than twice the data sample window. These thresholds were chosen because it would not be possible to infer such periods from the input data.

The results of the fit are estimates for m, A, λ and ϕ. The physical interpretations of all these parameters, respectively, are: the mean, the amplitude of the wave, the frequency (which can easily be converted to period, using period $= 24/\lambda$) and the time of day at which the wave attains its maximum value, a quantity known as the *acrophase.*

Note that there are 2 cases to consider when choosing the correct acrophase:

1. The wave has a period < 24 hours. This means there are multiple maximums in every 24h window

2. The wave has a period > 24 hours. This means that a maximum is not necessarily attained in every 24h window

To deal with option 1, we define the acrophase to be the time associated with the first peak during the sample window. For option 2, if there is a peak during our sample window then the associated time is defined as the acrophase. If there is no peak within our sample window, then we choose the peak that is associated with the acrophase closest to our sample window (so this can be before or after the sample window).

4 ClinCirc

In the combined approach, the Lomb–Scargle periodogram and the non–constrained cosinor estimate of period are used as a pre-processing filter to remove non-circadian signals before a constrained cosinor analysis is used to ascertain the characteristics of any detected circadian oscillation. Note that if the data are complete and uniformly spaced, the Lomb–Scargle periodogram is equivalent to the periodogram calculated using the Fast Fourier Transform. In ClinCirc, only if the maximum value of the periodogram occurs at 24 h is an unconstrained cosinor analysis performed. Next, only if the period estimate falls between 4h and $> 2 \times$ sample window length is the signal deemed to be circadian. Then, and only then, is a constrained cosinor analysis (period constrained between 18h–30h) performed to determine estimates for the amplitude, phase, and period. The period constraint is required because the Lomb–Scargle periodogram does not rule out signals with components in the higher harmonics (e.g., 12-hour period), provided

that the amplitudes of these components are less than those of the 24-hour component. Given the sparsity of the sampled data, these signals may be biologically significant, but will lead to periods that differ from 24 hours when fitting a single sinusoid. The lower limit is chosen as the mean of the fundamental (24 hours) and first harmonic (12 hours) and the upper limit is chosen so that the window is symmetric about 24 hours.

5 Gaussian process regression (GPR)

5.1 Using Gaussian processes to fit time series data

In this approach, we assume that our target time series $x(t)$ can be written as

$$x(t) = f(t) + \epsilon(t), \tag{2}$$

comprising a signal term $f(t)$ together with a noise term $\epsilon(t)$. Given N observations of the input (time) variable $X = (t_1, \ldots, t_N)^T$ and the output (*e.g.* gene expression) variable $\mathbf{y} = (x(t_1), \ldots, x(t_N))^T$, the regression task is to learn a model from this training data that can predict the output $\mathbf{y}_*$ from a new input $X_* = (t_1^*, \ldots, t_M^*)^T$.

Gaussian process regression (GPR) uses a Gaussian process as a prior for the function f in (2) – that is, we assume $f(t) \sim \mathcal{GP}(m(t), k(t,t'))$. A Gaussian process is a collection of random variables such that any finite collection of the random variables follows a multivariate normal distribution. It is characterised by a mean function $m(t) = \mathbb{E}(f(t))$ and a covariance function $k(t,t') = \mathbb{E}\left((f(t) - m(t))(f(t') - m(t'))\right)$. Thus, if we write $\mathbf{f} = (f(t_1), \ldots, f(t_N))^T$, then under this assumption, $\mathbf{f}$ is normally distributed with mean vector $m(X)$ and covariance matrix $K(X,X)$, where $K(X,X)_{ij} = k(t_i, t_j)$ – that is, $\mathbf{f} \sim \mathcal{N}(m(X), K(X,X))$. If is further assumed that the noise term $\epsilon(t)$ in (2) is independent of $f(t)$ and normally distributed with zero mean and standard deviation σ_n, that is $\epsilon(t) \sim \mathcal{N}(0, \sigma_n^2)$, it follows that the output variable $\mathbf{y}$ is distributed according to $\mathbf{y} \sim \mathcal{N}\left(m(X), K(X,X) + \sigma_n^2 I\right)$.

It can then be shown (see *e.g.* (5)) that conditioned on the training data $\{X, \mathbf{y}\}$, the function f evaluated at a new input X_*, which we write as $\mathbf{f}_*$, has the posterior distribution $\mathbf{f}_* | \{X, \mathbf{y}, X_*\} \sim \mathcal{N}\left(\bar{\mathbf{f}}_*, \mathrm{cov}(\mathbf{f}_*)\right)$, in which

$$\bar{\mathbf{f}}_* = K(X_*, X)\left(K(X,X) + \sigma_n^2 I\right)^{-1} \mathbf{y}$$

and

$$\mathrm{cov}(\mathbf{f}_*) = K(X_*, X_*) - K(X_*, X)\left(K(X,X) + \sigma_n^2 I\right)^{-1} K(X, X_*),$$

where $K(X_*, X)_{ij} = k(t_i^*, t_j)$, $K(X, X_*)_{ij} = k(t_i, t_j^*)$ and $K(X_*, X_*)_{ij} = k(t_i^*, t_j^*)$. The corresponding output variable $\mathbf{y}_*$ has the posterior distribution $\mathbf{y}_* | \{X, \mathbf{y}, X_*\} \sim \mathcal{N}\left(\bar{\mathbf{f}}_*, \mathrm{cov}(\mathbf{f}_*) + \sigma_n^2 I\right)$.

The covariance function $k(t,t')$ models the dependence between the function values at different time points t and t', and is referred to as the kernel of the Gaussian process. The choice of an appropriate kernel for a particular regression task is guided by domain-specific knowledge, such as the temporal patterns that are likely to be observed in the data (5). A commonly-used kernel is the radial basis function (RBF), which encodes the assumption that the correlation between two time points decays exponentially with the distance between them:

$$k(t,t') = \sigma_f^2 \exp\left(-\frac{(t-t')^2}{2\lambda^2}\right).$$

The kernel contains two hyperparameters: the signal variance σ_f^2 and the length scale λ. Together with the hyperparameter σ_n^2 of the noise process $\epsilon(t)$ in (2), these can be varied to improve the fit to a target timeseries. Given a chosen kernel k and hyperparameters

$\boldsymbol{\theta}$, a common method (and the method used here) for inferring the values of $\boldsymbol{\theta}$ is to maximise the log marginal likelihood of the training data

$$\log p(\mathbf{y}|X,\boldsymbol{\theta}) = -\frac{1}{2}\mathbf{y}^T K_{\mathbf{y}}^{-1}\mathbf{y} - \frac{1}{2}\log|K_{\mathbf{y}}| - \frac{N}{2}\log 2\pi, \tag{3}$$

where $K_{\mathbf{y}} = K(X,X) + \sigma_n^2 I$ (5).

5.2 GPR fits to circadian time series

In applying GPR to the circadian data (synthetic and experimental), we assumed a zero prior mean m and a covariance function k comprising the sum of a linear kernel (to model baseline drift), and a periodic kernel (a variant of the RBF kernel that can model rhythmic oscillations):

$$k(t,t') = \sigma_b^2 + \sigma_v^2(t-c)(t'-c) + \sigma_f^2 \exp\left(-\frac{2\sin^2(\pi|t-t'|/\tau)}{\lambda^2}\right). \tag{4}$$

The hyperparameters in this case are $\boldsymbol{\theta} = (\sigma_b, \sigma_v, c, \sigma_f, \lambda, \tau, \sigma_n)$, where σ_b^2, σ_v^2 and σ_f^2 are signal variances, c is an offset, λ is the length scale, τ is the period and σ_n^2 is the noise variance. Hyperparameters were fitted to z-score normalised training data by maximising the log marginal likelihood (3) using the Nelder-Mead simplex algorithm (6), as implemented by the MATLAB function `fminsearch`. To ensure that the global maximum was located in each case, the optimisation algorithm was initiated from 100 Latin hypercube samples generated within the following bounds:

$$\sigma_b = \sigma_v = c = 0,\ 0.001 \le \sigma_f, \lambda \le 10,\ 12 \le \tau \le 36,\ 0.001 \le \sigma_n \le 2 \text{ (simulated data)}$$
$$0.001 \le \sigma_b, \sigma_v, c, \sigma_f, \lambda \le 10,\ 18 \le \tau \le 30,\ 0.001 \le \sigma_n \le 2 \text{ (experimental data)}$$

For the fits to synthetic data, the parameters σ_b, σ_v, and c were restricted to 0 as this prevented the optimisation procedure from trying to fit a linear trend to the data, focusing instead on calibrating the periodic kernel hyperparameters. Preliminary optimisation runs demonstrated that if σ_b, σ_v, and c were allowed to vary, then correctly locating the global maximum became extremely difficult, and typically resulted in several of the hyperparameters taking extreme values. This is because the hyperparameter space becomes populated with 'neutral zones' in which the optimiser can take large steps without improving the quality of the fit.

5.3 GPR-based circadian rhythmicity criterion

Given the maximum likelihood estimate $\hat{\boldsymbol{\theta}} = \left(\hat{\sigma}_b, \hat{\sigma}_v, \hat{c}, \hat{\sigma}_f, \hat{\lambda}, \hat{\tau}, \hat{\sigma}_n\right)$ of $\boldsymbol{\theta}$ obtained as described above, a time series $x(t)$ was then classified as circadian if the kernel hyperparameter values satisfied the following inequalities:

$$\sigma_v < 0.1,\ \sigma_f > 0.1,\ \lambda > 0.5,\ 20 < \tau < 28,$$

The bounds on σ_v and σ_f ensured that the signal term $f(t)$ in (2) was not dominated by baseline drift, whilst the bound on the length scale λ excluded very high frequency variations in expression level. The bounds on τ reflected a broad definition of circadian rhythmicity. All time series that satisfied this criterion were observed to have self-sustained oscillations with a roughly circadian period, validating the bounds chosen.

5.4 Calculation of posterior distributions and circadian metrics

Posterior distributions $\{\bar{\mathbf{f}}_*, \mathbf{y}_*\}$ were computed using custom-written software in MATLAB (these will be made available following publication from `https://github.com/oeakman`). Acrophases and amplitudes were extracted from GPR fits by evaluating the posterior mean $\bar{\mathbf{f}}_*$ on a finely sampled mesh X_*, and applying the MATLAB function `findpeaks`. 95% confidence intervals were computed as $(\bar{\mathbf{f}}_*)_i \pm 1.96\sigma_i$, where $\sigma_i^2 = (\mathrm{cov}(\mathbf{f}_*))_{ii} + \sigma_n^2$ (5).

5.5 Application of the Matérn kernel for detecting periodicities

An alternative approach to quantifying if a signal is circadian using GPR was suggested by (7). They focus on applying harmonic analysis in reproducing kernel Hilbert spaces (RKHS). Specifically, they decompose an RKHS into a subspace of periodic kernels and combine this with its orthogonal complement to produce a subspace of aperiodic sub-kernels. It is shown that the Matérn family of kernels can be analytically decomposed in this fashion and this decomposition allows the quantification of the periodicity of a signal. The final kernel applied uses a Matérn 3/2 kernel plus a delta function to capture observational noise:

$$k(t,t') = \sigma_p^2 k_p(t,t') + \sigma_a^2 k_a(t,t') + \tau^2 \delta(t,t'),$$

where k_p is the periodic sub-kernel and k_a is the aperiodic sub-kernel. Once the GPR has been fit with this kernel, the periodic and aperiodic sub-kernels can be used to quantify the periodicity of the time series. The criterion is based on the ratio of signal variance explained by the sub-kernels. Explicitly, let R be a random variable defined on the input space, and y_p, y_a be the periodic and aperiodic components of the signal y. The periodicity ratio is defined as

$$S = \frac{\mathrm{Var}\left[y_p(R)\right]}{\mathrm{Var}\left[y_p(R) + y_a(R)\right]}.$$

It must be noted that S can not be interpreted as the percentage of periodicity in the signal since $\mathrm{Var}\left[y_p(R) + y_a(R)\right] \neq \mathrm{Var}\left[y_p(R)\right] + \mathrm{Var}\left[y_a(R)\right]$. As such, this ratio can exceed 1.

To apply this method to detect if a signal is circadian, the hyperparameters are constrained as $\lambda \in [20, 28]$ (the period); $\sigma_p, \sigma_a \geq 0$ (variance of the periodic and aperiodic sub-kernels); $l_p, l_a \in [10, 60]$ (the length scales of the periodic and aperiodic sub-kernels) and $\tau^2 \in \left[10^{-5}, 0.75\right]$. To try limit the impact of local minima, 50 random restarts are performed for each optimisation. Finally, since the periodicity ratio S is a random variable, the expectation is approximated using the mean of 1000 realisations.

The code provided by the authors of (7) in their supplementary materials was used to implement this method. No strict threshold was given on what value of S indicates a periodic signal, but based on testing the sensitivity and specificity of the method on synthetic data, a cut-off of 0.7 was used.

6 Generating synthetic data

The performance of each method was analysed using synthetic data generated with a 4-hour sampling frequency over both 24-hour and 48-hour sample windows.

We considered two key metrics for evaluating the performance of each algorithms: specificity and sensitivity. In the context of circadian analysis, the specificity indicates the proportion of non-circadian signals that an algorithm will correctly determine are not circadian, whilst sensitivity is the proportion of genuine circadian signals that are correctly identified as circadian.

6.1 Specificity assessment

In order to determine how well each algorithm could identify non-circadian signals, data were simulated from a straight line as has previously been suggested (8). Specifically, data were generated using the model

$$X_t = mt,$$

where $m \in (-0.05, 0]$. This model was used to generate two datasets: one sampled every 4 hours for 24 hours, and one sampled every 4 hours for 48 hours. Each dataset contained 5000 synthetic waveforms.

6.2 Sensitivity assessment

To assess the sensitivity of each algorithm, data were generated from a cosine wave following the simultaneous addition of noise to the amplitude, period, and acrophase. The model used to generate synthetic signals was a two-step process. Firstly, a random amplitude and acrophase were selected for a cosine wave:

$$\begin{aligned} A &\sim Unif[0.1, 10], \\ \varphi &\sim Unif[0, 24], \\ \gamma &\in \{-0.05, 0.05\}. \end{aligned}$$

That is, the amplitude is uniformly sampled from 0.1 to 10, the acrophase is uniformly sampled from [0, 24], and a linear trend was randomly selected to be increasing or decreasing using gradients previously suggested (8). For each signal, once the above parameters had been selected, each synthetic data point was generated from the model

$$X_t = M + A\cos\left(\frac{2\pi t}{\text{period}} - \Phi\right) + \varepsilon(t) + \gamma t,$$

where

$$\begin{aligned} M &= \text{MESOR} = 1, \\ \text{period} &\sim Unif[24 - \alpha_{\text{per}}, 24 + \alpha_{\text{per}}], \\ \Phi &\sim Unif[\varphi - \alpha_{\text{phase}}, \varphi + \alpha_{\text{phase}}], \\ \varepsilon(t) &\sim N(0, \alpha_{\text{amp}} \cdot A). \end{aligned}$$

Hence, every time a point was generated from a signal, the period, amplitude and acrophase had added noise. There are three noise parameters, $\alpha_{\text{per}}, \alpha_{\text{amp}}, \alpha_{\text{phase}}$, that can be tuned to increase or decrease the amount of noise added. The maximum noise level we used, termed the 40% noise level, was parameterised by $\{\alpha_{\text{per}}, \alpha_{\text{amp}}, \alpha_{\text{phase}}\} = \{4, 0.4, 1\}$, so that the period could vary between 20 − 28 hours, the variance of the noise added to the amplitude was equal to 40% of the amplitude of the wave, and the phase could vary by ±1hour. Any lower level of noise was scaled proportionally i.e., 20% noise has the parameters $\{\alpha_{\text{per}}, \alpha_{\text{amp}}, \alpha_{\text{phase}}\} = \{2, 0.2, 0.5\}$. A selection of wave forms generated using this procedure can be seen in (**Supp. Fig. 1**), ranging from 0% noise (a perfect sinusoid) up to 40% noise in 10% increments. Data were sampled every 4 hours over both 24 hours and 48 hours to reflect the clinical data and 5000 synthetic signals were generated for each dataset.

7 Benchmarking performance using biological data

The performance of the algorithms were also evaluated using biological data. For this purpose bioluminescence data from lung slices obtained from *PER2*::Luc mice was analysed. Initial recordings were made every 10 minutes over 72 hours from three different lung slices. The expression levels have an amplitude that dampens over the course of 72 hours, but it is clear they have a roughly 24 hour period To adapt these data for benchmarking, the traces were initially split into either 24- or 48-hour sampling periods. Next, the data were down-sampled to be measured once every 4 hours. Each algorithm was applied to these datasets and assessed based on how many of the waveforms it correctly identified as circadian.

The biological data was then used for a second set of benchmarks, this time focusing on assessing the accuracy of parameter estimation. For this task, Gaussian noise was added to the traces. That is, for every data point (X_t, Y_t), a new data observation $\hat{Y}_t = Y_t + \varepsilon(t)$, was generated. Explicitly, $\varepsilon(t)$ has the distribution

$$\varepsilon(t) \sim N(0, \alpha\hat{A}),$$

where α was varied from 0 to 0.4 in steps of 0.1 and $\hat{A} = 0.5(\max(Y_t) - \min(Y_t))$, a non-parametric approximation of the amplitude. This resulted in a new data set of data points, $(X_t, \hat{Y}_t)$, referred to as a "noisy" dataset. Using this method, 250 noisy datasets were generated for each noise level (i.e., for each value of α). A "ground truth" for the parameter estimates was then calculated using the original waveforms, sampled every 10 minutes. To assess the accuracy of the parameters estimates, the prediction for amplitude, period, and phase on the noisy datasets was compared to ground truth estimates.

References

1. J. T. VanderPlas, "Understanding the lomb–scargle periodogram," *The Astrophysical Journal Supplement Series*, vol. 236, no. 1, p. 16, 2018.

2. J. D. Scargle, "Studies in astronomical time series analysis. ii-statistical aspects of spectral analysis of unevenly spaced data," *The Astrophysical Journal*, vol. 263, pp. 835–853, 1982.

3. M. Suveges, "False alarm probability based on bootstrap and extreme-value methods for periodogram peaks," in *ADA7-Seventh Conference on Astronomical Data Analysis*, vol. 1, 2012, p. 16.

4. G. Cornelissen, "Cosinor-based rhythmometry," *Theoretical Biology and Medical Modelling*, vol. 11, no. 1, pp. 1–24, 2014.

5. C. K. Williams and C. E. Rasmussen, *Gaussian processes for machine learning.* MIT press Cambridge, MA, 2006, vol. 2, no. 3.

6. J. C. Lagarias, J. A. Reeds, M. H. Wright, and P. E. Wright, "Convergence properties of the nelder–mead simplex method in low dimensions," *SIAM Journal on optimization*, vol. 9, no. 1, pp. 112–147, 1998.

7. N. Durrande, J. Hensman, M. Rattray, and N. D. Lawrence, "Detecting periodicities with gaussian processes," *PeerJ Computer Science*, vol. 2, p. e50, 2016.

8. G. Wu, J. Zhu, J. Yu, L. Zhou, J. Z. Huang, and Z. Zhang, "Evaluation of five methods for genome-wide circadian gene identification," *Journal of biological rhythms*, vol. 29, no. 4, pp. 231–242, 2014.
